# Identification of different classes of genome instability suppressor genes through analysis of DNA damage response markers

**DOI:** 10.1093/g3journal/jkae064

**Published:** 2024-03-25

**Authors:** Bin-Zhong Li, Richard D Kolodner, Christopher D Putnam

**Affiliations:** Ludwig Institute for Cancer Research, San Diego Branch, La Jolla, CA 92093-0669, USA; Ludwig Institute for Cancer Research, San Diego Branch, La Jolla, CA 92093-0669, USA; Department of Cellular and Molecular Medicine, University of California San Diego, La Jolla, CA 92093-0669, USA; Moores-UCSD Cancer Center, University of California San Diego, La Jolla, CA 92093-0669, USA; Institute of Genomic Medicine, University of California San Diego, La Jolla, CA 92093-0669, USA; Ludwig Institute for Cancer Research, San Diego Branch, La Jolla, CA 92093-0669, USA; Department of Medicine, University of California San Diego, La Jolla, CA 92093-0669, USA

**Keywords:** DNA damage signaling, DNA repair, genome instability

## Abstract

Cellular pathways that detect DNA damage are useful for identifying genes that suppress DNA damage, which can cause genome instability and cancer predisposition syndromes when mutated. We identified 199 high-confidence and 530 low-confidence DNA damage-suppressing (DDS) genes in *Saccharomyces cerevisiae* through a whole-genome screen for mutations inducing Hug1 expression, a focused screen for mutations inducing Ddc2 foci, and data from previous screens for mutations causing Rad52 foci accumulation and Rnr3 induction. We also identified 286 high-confidence and 394 low-confidence diverse genome instability-suppressing (DGIS) genes through a whole-genome screen for mutations resulting in increased gross chromosomal rearrangements and data from previous screens for mutations causing increased genome instability as assessed in a diversity of genome instability assays. Genes that suppress both pathways (DDS+ DGIS+) prevent or repair DNA replication damage and likely include genes preventing collisions between the replication and transcription machineries. DDS+ DGIS− genes, including many transcription-related genes, likely suppress damage that is normally repaired properly or prevent inappropriate signaling, whereas DDS− DGIS+ genes, like *PIF1*, do not suppress damage but likely promote its proper, nonmutagenic repair. Thus, induction of DNA damage markers is not a reliable indicator of increased genome instability, and the DDS and DGIS categories define mechanistically distinct groups of genes.

## Introduction

Cells have sensitive mechanisms to identify and signal the presence of DNA damage. Mutations affecting these pathways have been linked to cancer predisposition syndromes in humans and increased genome instability in both model organisms and human cells and tumors (Vogelstein and Kinzler 2004; Friedberg *et al.* 2013; Carbone *et al.* 2020; Sharma *et al.* 2020). Markers for the presence of DNA damage have been widely exploited as in vivo sensors for increased levels of DNA damage (Sun *et al.* 1996; Maser *et al.* 1997; Nelms *et al.* 1998; Zhong *et al.* 1999; Downs *et al.* 2000; Paull *et al.* 2000; Melo *et al.* 2001; Fernandez-Capetillo *et al.* 2002; Wang *et al.* 2002; Lou *et al.* 2003; Stewart *et al.* 2003).

One important class of DNA damage response (DDR) markers includes targets of the DNA damage checkpoint. In *Saccharomyces cerevisiae*, the DNA damage checkpoint is triggered by a protein kinase cascade comprised of Tel1^ATM^ or Mec1^ATR^-Ddc2^ATRIP^, Rad53^CHEK2^, and Dun1 (Supplementary Fig. 1; Putnam, Jaehnig, *et al.* 2009; Ciccia and Elledge 2010; Lanz *et al.* 2019). Markers of checkpoint activation include (1) the accumulation of phosphorylated forms of histone H2A (the *S. cerevisiae* equivalent of γ-H2AX), Rad53^CHEK2^, RPA, Rad54, and Rad55 (Sun *et al.* 1996; Bashkirov *et al.* 2000, 2006; Downs *et al.* 2000; Herzberg *et al.* 2006; Smolka *et al.* 2007; Chen *et al.* 2010; Lanz *et al.* 2021) and (2) changes in the levels of ribonucleotide reductase (RNR)-related proteins, including Rnr1–4, Sml1, and Hug1 (Supplementary Fig. 1; Huang *et al.* 1998; Zhao *et al.* 2001). Together, these responses trigger DNA damage checkpoint activation, which can arrest cells at the G2/M cell cycle transition and increase the deoxyribonucleotide (dNTP) levels to promote DNA replication and DNA repair. Induction of dNTP levels is the essential function of the Mec1^ATR^-Ddc2^ATRIP^ and Rad53^CHEK2^ proteins in *S. cerevisiae* (Desany *et al.* 1998; Huang *et al.* 1998; Zhao *et al.* 1998; Basrai *et al.* 1999; Lee and Elledge 2006; Zhang *et al.* 2006; Lee *et al.* 2008; Wu and Huang 2008; Meurisse *et al.* 2014).

A second important class of the DDR markers includes proteins that form discrete nuclear foci in response to DNA damage. Nuclear foci of DNA repair proteins that form and colocalize with double-strand breaks (DSBs) have been identified, including the Mre11-Rad52-Xrs2 complex, Rad52, Rad51, Rad54, Rad55, Rad59, Rdh54, and RPA (Maser *et al.* 1997; Nelms *et al.* 1998; Melo *et al.* 2001; Lisby *et al.* 2003, 2004; Lisby and Rothstein 2009). These foci have been used to elucidate an ordered assembly pathway for DSB repair proteins in *S. cerevisiae* (Lisby *et al.* 2004). Foci have also been observed for other DNA repair proteins as well as components of the DNA damage checkpoint, such as Ddc2^ATRIP^ and Ddc1^RAD9A/RAD9B^ (Melo *et al.* 2001; Makhnevych *et al.* 2009; Hombauer *et al.* 2011; Tkach *et al.* 2012; Yimit *et al.* 2015).

Despite their utility, the consistency between these different DDR markers and their correlation with the results of genome instability assays has not been systematically investigated. Here, we have performed genome-wide screens for mutations in *S. cerevisiae* that induce Hug1 expression and induce the accumulation of gross chromosomal rearrangements (GCRs) detected using the duplication-mediated GCR assay (Putnam, Hayes, *et al.* 2009) as well as a screen for mutations causing increased Ddc2 foci using a focused set of query mutations. We have combined these data with data from 3 previous genome-wide DDR marker screens (Alvaro *et al.* 2007; Hendry *et al.* 2015; Styles *et al.* 2016), the results of 14 different studies identifying mutations that cause increased genome instability as assessed in a range of different genome instability assays (Chen and Kolodner 1999; Yuen *et al.* 2007; Andersen *et al.* 2008; Putnam, Hayes, *et al.* 2009; Chan and Kolodner 2011; Stirling *et al.* 2011; Zhang *et al.* 2012, 2013; Putnam *et al.* 2016; Nene *et al.* 2018; Srivatsan *et al.* 2019; Novarina *et al.* 2020), and systematic screens for mutations that alter the cell cycle distribution in log-phase cells (Koren *et al.* 2010; Hoose *et al.* 2012; Soifer and Barkai 2014). We used these data to define sets of 199 high-confidence and 530 low-confidence genes that suppress DNA damage levels and 286 high-confidence and 394 low-confidence genes that suppress genome instability during unperturbed cell growth; high-confidence genes are those that were identified in multiple assays. Genes that act in DNA damage suppression and genome instability suppression (DDS+ DGIS+) are strongly linked to preventing or repairing endogenous damage arising during DNA replication. Remarkably, we also identified many high-confidence genes that only suppress DNA damage (DDS+ DGIS−) or only suppress genome instability (DDS− DGIS+). The DDS+ DGIS− genes may suppress DNA damage that is only rarely misrepaired to cause genome instability or may suppress inappropriate signaling, whereas the DDS− DGIS+ genes do not suppress damage but likely promote the correct repair of damage when it occurs. Together, our results provide mechanistic insights into the prevention and processing of spontaneous DNA damage in unperturbed cells.

## Materials and methods

### Bait strain construction

The bait strain containing the *HUG1-EGFP* marker was created by integrating a *EGFP-hphNT1* cassette amplified by PCR onto the 3′ end of the *HUG1* coding sequencing in the strain RDKY7635 (Putnam *et al.* 2016) to create RDKY8174 (*MATα his3Δ200  hom3-10 leu2Δ0 trp1Δ63 ura3Δ0 lyp1::TRP1 cyh2-Q38K iYFR016C::P*_*MFA1*_*-LEU2 can1::P*_*LEU2*_*-NAT yel072w::CAN1-URA3  HUG1-EGFP.hphNT1*); note that this strain contains the dGCR assay markers. The bait strain containing the *DDC2-EGFP* marker was created by a multistep process (see Supplementary Methods S1) to introduce markers required for systematic mating, the NUP49-mCherry fluorescent marker, and the Ddc2-EGFP fluorescent marker to create RDKY8934 (*MATα his3Δ200 leu2Δ0 trp1Δ63 ura3Δ0 can1 cyh2-Q38K iYFR016C:P*_*MFA1*_*-URA3 NUP49-mCherry.hphNT1 DDC2-EGFP.HIS3MX6*).

### Systematic genetic crosses

The *HUG1-EGFP* containing bait strain RDKY8174 was crossed twice to a selected set of the BY4741  *MAT***a** strains in *S. cerevisiae* deletion collection (*MAT***a**  *his3Δ1 leu2Δ0 met15Δ0 ura3Δ0*), which was enriched for genes predicted to be involved in genome stability (Putnam *et al.* 2012), and once to the entire collection of BY4741  *MAT***a** strains using a RoToR pinning robot (Singer Instruments). The *DDC2-EGFP.HIS3MX6* containing bait strain was crossed to a set of 468 BY4741  *MAT***a** deletion strains containing mutations selected as causing increased Hug1-EGFP expression or increased GCR rates or the *leu2Δ::kanMX4* control mutation. Systematic mating followed a modified synthetic genetic array (SGA) protocol (Tong and Boone 2006) involving multiple steps: diploid selection, sporulation, diploid killing, and haploid selection (see Supplementary Methods S1). Due to the different markers in the two bait strains, different selective media were used at some steps (see Supplementary Methods S1).

### DNA content analysis

Cell cycle analysis was conducted as previously described (Vanoli *et al.* 2010). In brief, 1 × 10^7^ cells were collected by centrifugation and resuspended in 70% ethanol for 16 h. Cells were then washed in 0.25 M Tris-HCl (pH 7.5), resuspended in the same buffer containing 2 mg/mL of RNaseA (Sigma Aldrich), incubated at 37°C for at least 1 h, and then treated overnight with proteinase K (1 mg/mL; Sigma Aldrich) at 37°C. Cells were collected by centrifugation, resuspended in 200 mM Tris-HCl (pH 7.5) buffer containing 200 mM NaCl and 80 mM MgCl_2_, and stained in the same buffer containing 1 µM SYTOX green (Invitrogen). Samples were then diluted 10-fold in 50 mM Tris-HCl (pH 7.8), sonicated in a Cole-Palmer 8891 Water Bath Sonicator, and analyzed using a Becton Dickinson FACScan instrument. This FACS analysis verified that all the strains used for analysis in experiments from this study were haploids.

### Measurement of Hug1-EGFP levels

To measure Hug1-EGFP expression, logarithmic-phase cultures were grown in YPD medium in 96 well plates and centrifuged, and the cells were resuspended in 100-µL sterile water and sonicated in a Cole-Palmer 8891 Water Bath Sonicator. The cells were then analyzed using a BD LSR Fortessa analytical cytometer with a High-Throughput Sampler. Excitation was at 488 nm, and the fluorescence signal was collected through a 505-nm long-pass filter and a HQ510/20 band-pass filter (Chroma Technology Corp). For each sample, 30,000 events were recorded. The mean value of the GFP fluorescence in each sample was calculated using FlowJo software and normalized to the mean GFP value of wild-type cells.

### Measurement of Ddc2-GFP foci

Cells were grown in a complete synthetic medium (Sigma Aldrich) to log phase and examined by live imaging using an Olympus BX43 fluorescence microscope with a 60× 1.42 PlanApo *N* Olympus oil-immersion objective. GFP fluorescence was detected using a Chroma FITC filter set and captured with a Qimaging QIClick CCD camera. Nuclear boundaries were identified by Nup49-mCherry fluorescence, and only Ddc2 foci contained within nuclei were scored. Images were analyzed using Meta Morph Advanced 7.7 imaging software, keeping processing parameters constant within each experiment.

### Cell cycle distribution data analysis

The cell cycle distributions of log-phase cells for the *MAT***a**  BY4741 haploid deletion collection and the homozygous diploid BY4743 deletion collection were previously published by others (Koren *et al.* 2010; Hoose *et al.* 2012; Soifer and Barkai 2014). For the diploid deletion collection data, the published supplement containing cell cycle parameters from the Dean–Jett–Fox model (Fox 1980) as implemented in FlowJo was used (Hoose *et al.* 2012). For the haploid deletion collection data, FACS data were downloaded from the FlowRepository (Spidlen *et al.* 2012) and were analyzed using the Dean–Jett–Fox model in FlowJo version 10 (FlowJo 2019). Analyses of these data to remove aggregates, merge multiple observations, and project onto ternary plots are described in Supplementary Methods S1.

### Genome-wide GCR screen

Haploid cells resulting from the cross of RDKY8174, which contains the dGCR assay markers, with the BY4741 deletion collection were grown in YPD in 96-well plates and patched in triplicate on YPD plates using a Singer RoToR robot. Patched YPD plates were then replica plated onto GCR selection medium plates [6.7-g yeast nitrogen base (Fisher Scientific), 2-g CSM-Arg-Ura dropout mix (US Biological), 60-mg uracil (Sigma Aldrich), 1-g 5-fluoroorotic acid (US Biological), 60-mg L-canavanine sulfate (Sigma Aldrich), 20-g glucose (Fisher Scientific), and 24-g bacto-agar (Fisher Scientific) per liter]. After 3–4 days of growth, the GCR selection medium plates were photographed and the number of papillae in each patch was counted. For each set of 3 plates, the distribution of papillae per patch was determined and fitted to a normal distribution. The *P*-values for each patch were then calculated and then a combined *P*-value for each mutant was determined using Fisher's sumlog procedure as implemented in R. Using a false discovery rate (FDR) of 0.05, 197 mutations of 193 genes were identified as causing increased GCRs. These hits were then rescreened by manual repatching in triplicate onto YPD plates and rescreening by replica plating onto GCR selective medium plates and counting papillae. A cutoff ratio, 1.8, for the fold increase relative to the average number of papillae per patch was determined by maximizing the Youden index using a set of 94 true positive mutations and 921 true negative mutations from previous studies. This cutoff identified 52 significant hits, 24 of which were previously identified as causing increased GCR rates in quantitative assays and 6 of which were previously identified as not causing increased GCR rates in quantitative assays. Selected mutations were then reconstructed in RDKY7635 (systematic dGCR assay; *MATα hom3–10 ura3Δ0 leu2Δ0 trp1Δ63 his3Δ200 lyp1::TRP1 cyh2-Q38K iYFR016C::P*_*MFA1*_*-LEU2 can1::P*_*LEU2*_*-NAT yel072w::CAN1-URA3*), and GCR rates were measured using published methods (Srivatsan *et al.* 2018).

### Quantification and statistical analysis

**Fig. 1.**
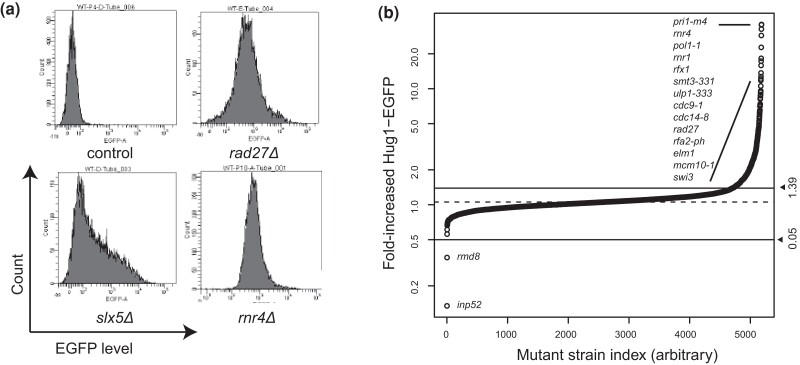
Distribution of Hug1-EGFP levels. a) Histograms of Hug1-EGFP levels in individual cells from log-phase cultures of the control (*leu2Δ*) strain and 3 mutant strains with increased Hug1-EGFP levels. b) Distribution of the fold-increased Hug1-EGFP levels for all mutants analyzed. The dashed line is the center of the fitted normal distribution, and solid lines are FDR < 0.05 significance cutoffs (Supplementary Fig. 3c and d).

**Fig. 2.**
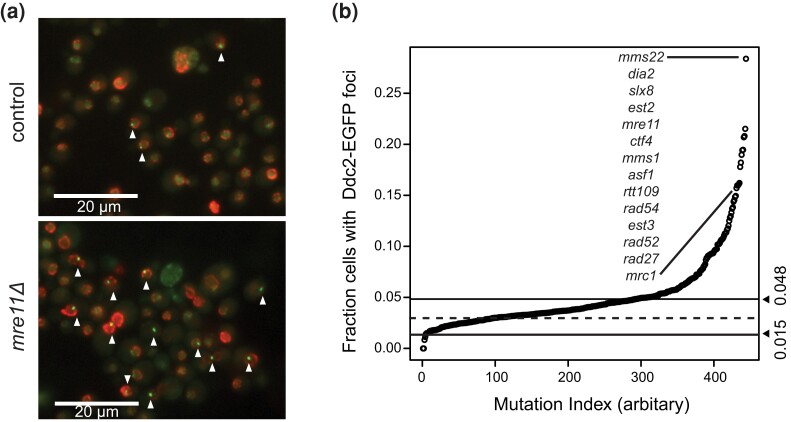
Induction of Ddc2-EGFP foci in mutant strains. a) Representative images of Ddc2-EGFP foci (triangles) for the control (*leu2Δ*) and *mre11Δ* strains. b) Distribution of the fraction of cells with Ddc2-EGFP foci. The dashed horizontal line is the average of the *leu2Δ* control strain measurements, and the solid horizontal lines correspond to the 95 and 5% percentiles of the control strain measurements.

All statistical analyses were performed with R version 4.1.1. Significance for the Hug1-EGFP expression data was performed by fitting to a Gaussian distribution as described in the main text, Fig. 1, and Supplementary Fig. 1. Significance for the Ddc2-GFP foci data was performed using 95% cutoffs of the wild-type data as described in the main text and Fig. 2. Significance for the cell cycle distribution was performed as described in the Supplemental Methods based on the two-independent Gaussian error model.

## Results

### Identification of genes suppressing Hug1-EGFP induction

Fluorescence-activated cell sorting (FACS) determines fluorescence intensity on a per-cell basis, making it suitable to monitor fluorescence responses even in strains with a slow growth phenotype. To identify mutations inducing DNA damage signaling, we first constructed a strain containing the *HUG1-EGFP* marker. We found that the *HUG1-EGFP* marker gave the largest change in expression upon hydroxyurea treatment in preliminary FACS experiments when compared to 3 other potential markers (Supplementary Fig. 2), and previous studies demonstrated that the *HUG1* promoter is responsive to a wide variety of DNA-damaging agents, but not other types of cellular stress (Benton *et al.* 2007; Ainsworth *et al.* 2012). We crossed this strain to the *S. cerevisiae*  BY4741 deletion collection using a SGA protocol (Supplementary Fig. 3a; Tong and Boone 2006; Putnam *et al.* 2016). For downstream analyses, we added a BY4741  *leu2Δ::kanMX4* control strain to the deletion collection by generating a strain in which the BY4741  *leu2Δ0* deletion was replaced with a *leu2Δ::kanMX4* deletion. Unperturbed log-phase cultures of the resulting *MAT***a** haploid strains that contained *HUG1-EGFP* and the gene deletion of interest were analyzed by high-throughput FACS for both DNA content and EGFP fluorescence. We used the DNA content measurements to identify isolates with diploid DNA content or with a mixture of cells with haploid and diploid DNA content; data from these problematic isolates were excluded from our analyses. Fold Hug1-EGFP induction levels were calculated by normalizing the average EGFP fluorescence for each strain to the fluorescence of control strains from the same experiment (Fig. 1a; Supplementary Table 1). Because subsets of the deletion collection were independently mated and measured up to 3 times, 4,791 deletion mutants derived from 5,781 independently generated strains were analyzed; repeated measurements from independent biological isolates were highly correlated (Supplementary Fig. 3b).

We combined the Hug1-EGFP expression data for the 4,791 deletion mutants determined here with our previous data for 394 strains with hypomorphic mutations in essential genes (Fig. 1b; Srivatsan *et al.* 2019). The resulting fold Hug1-EGFP values were normally distributed with a mean of 1.06 and a standard deviation of 0.13, which indicates that most mutations do not substantially alter Hug1 expression (Supplementary Fig. 3c). More strains in the experimental distribution had increased Hug1 expression than were predicted by a fitted normal distribution, indicating the presence of mutations that induce Hug1 expression during unperturbed growth (Supplementary Fig. 3c and d). Using a FDR of 0.05, 479 mutations affecting 416 genes were identified as causing increased Hug1-EGFP expression (1.39- to 35.8-fold induction; Supplementary Table 1). Details of these genes are described below.

### Induction of Ddc2 foci imperfectly correlates with Hug1 induction

Hug1 induction is a downstream readout of the Mec1 DNA damage checkpoint (Supplementary Fig. 1; Huang *et al.* 1998; Basrai *et al.* 1999). We therefore examined an upstream marker of Mec1 activation, the accumulation of Ddc2 foci (also called Lcd1 foci; Melo *et al.* 2001; Waterman *et al.* 2019). We constructed a *MATα* bait strain expressing a functional Ddc2-EGFP fusion protein (Supplementary Fig. 4a) and the Nup49-mCherry nuclear pore fusion protein, which allows for scoring of only nuclear Ddc2 foci. We crossed this bait strain using SGA (Supplementary Fig. 4b) to a focused set of 469 BY4741  *MAT***a** strains enriched for deletion mutations that induced Hug1 expression. The resulting 444 recovered haploid strains were grown to log phase and analyzed by fluorescence microscopy to determine the fraction of cells containing nuclear Ddc2 foci (Fig. 2a; Supplementary Table 2). Because this focused screen was biased for mutations inducing DNA damage, we assessed significance thresholds using the 5th and 95th percentile of the *leu2Δ* control strain observations. Only 4 of the strains had significantly reduced levels of Ddc2 foci (fraction of cells with Ddc2 foci < 0.015): *npl3Δ*, *ydjlΔ*, *dus1Δ*, and *mec1Δ sml1Δ*. In contrast, 153 strains had significantly increased levels of Ddc2 foci (fraction of cells with Ddc2 foci > 0.048; Fig. 2b), and many, but not all, of these mutant strains were also identified in the Hug1 induction screen as having increased levels of Hug1 expression (see below).

### DDR screens implicate DNA replication errors as a major source of spontaneous DNA damage

**Fig. 3.**
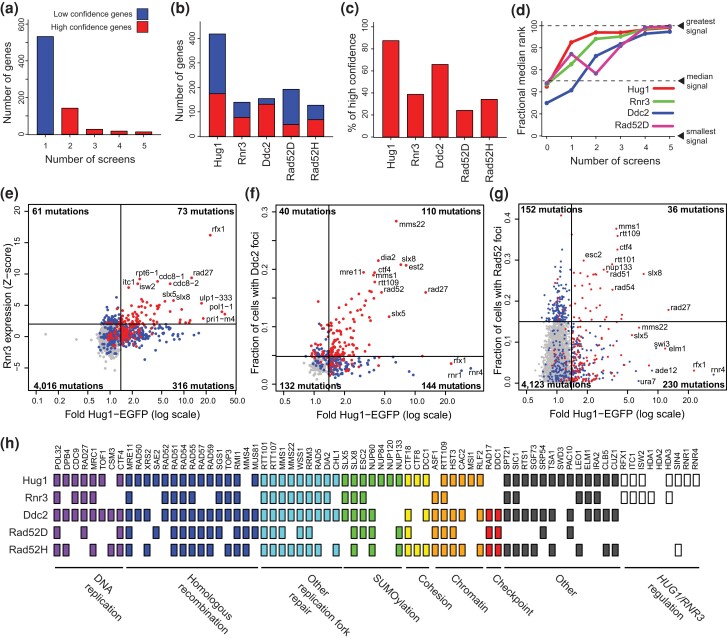
Comparison of DDR marker screens. a) Histogram of the number of screens genes were identified in. High-confidence genes were identified in multiple screens. Low-confidence genes were identified in one screen. b) Total number of genes identified in each screen. c) Percentage of high-confidence DDR genes identified in each screen, which was calculated by dividing the number of high-confidence genes identified in each screen b) by the total count of 199 high-confidence DDR genes. d) Mutations of genes identified in more screens cause stronger effects. Mutations are grouped by the number of screens the affected gene was identified in, and the screen results are rank ordered by the magnitude of the signal in the quantitative assays (100% = strongest signal and 0% = weakest signal). The fractional median rank is plotted for each group in each screen (red = Hug1, green = Rnr3, blue = Ddc2, and purple = Rad52D). e) Plot of the Rnr3 expression *Z*-score vs the fold increase in Hug1-EGFP expression. Lines are significance thresholds; the *Z*-score threshold of 2 was used in the original study (Hendry *et al.* 2015). f) The fraction of cells with Ddc2-EGFP foci vs the fold Hug1-EGFP expression plotted as for d). g) The fraction of diploid cells with Rad52 foci vs the fold Hug1-EGFP expression plotted as for d). The threshold of 0.15 was one used in the original study (Alvaro *et al.* 2007). h) A subset of high-confidence DDS genes, including all genes identified at least 3 times, grouped by function.

To better understand the mutations causing induced Hug1 expression and increased Ddc2 foci levels, we compared the data generated here with data from previous screens for mutations affecting DNA damage responses. These screens included (1) induction of Rnr3 expression (Hendry *et al.* 2015); (2) induction of Rad52 foci in diploid strains, which are intermediates during homologous recombination of DSBs and replication fork damage (Lisby *et al.* 2001) (Rad52D screen; Alvaro *et al.* 2007); and (3) induction of Rad52 foci in haploid strains (Rad52H screen; Styles *et al.* 2016). Together, these screens evaluated 5,976 mutations and identified 729 DNA damage-suppressing (DDS) genes, including 199 high-confidence DDS genes identified in multiple screens and 530 low-confidence DDS genes identified in only 1 screen (Fig. 3a–c; Supplementary Table 3). In general, mutations in genes identified by multiple screens tended to cause larger induction responses (Fig. 3d), consistent with the idea that mutations causing high levels of DNA damage are robustly identified in multiple screens using different DNA damage response readouts. Because only some of the screens analyzed alleles of essential genes and because some strains may not have been recovered from crosses, some low-confidence genes are likely to be bona fide DDS genes as these factors reduce the possibility that a gene could be scored as a high-confidence DDS gene.

We directly compared the quantitative data from the Hug1 screen with the data from the Rnr3 (Fig. 3e), Ddc2 foci (Fig. 3f), and Rad52D foci screens (Fig. 3g). Consistent with the similarities of *HUG1* and *RNR3* regulation (Supplementary Fig. 1; Huang *et al.* 1998; Basrai *et al.* 1999), we observed a modest correlation between the data from the Hug1 and Rnr3 screens (Fig. 3e); 4,466 mutations were tested in both assays, and 73 mutations induced both markers, whereas 316 mutations only induced Hug1 and 61 mutations only induced Rnr3. This modest correlation may reflect the fact that induction of Rnr3 was a less sensitive DNA damage sensor than induction of Hug1 (Supplementary Fig. 1). Comparison with the Ddc2 screen results distinguished 2 groups of Hug1-inducing mutations among the 434 mutations tested in both assays. For mutations identified in both the Hug1 and Ddc2 screens (Fig. 3f, upper right quadrant; 110 mutations), the damage marker levels were correlated, suggesting that the mutations cause both increased DNA damage and increased Mec1-Ddc2-Hug1 signaling. For the uncorrelated mutations that only induced Hug1 (Fig. 3f, lower right quadrant; 144 mutations), these mutations may induce Hug1 through mechanisms independent of increased DNA damage (Basrai *et al.* 1999). The results from the Rad52D foci screen had relatively poor correlation with the results from the Hug1 screen; of the 4,541 mutations measured in both assays, only 36 mutations caused significant induction of both Hug1 expression and Rad52 foci (Fig. 3g). This poor correlation may result from the fact that increased Rad52 foci can be caused by defects resulting in (1) increased DNA damage, (2) increased processing of normal levels of endogenous DNA damage by HR, and (3) delays in the turnover of HR intermediates, the latter two of which may not be recognized as increased DNA damage. Other foci markers used to monitor the DNA damage response, including BRCA1, BRCA2, RAD51, MDC1, 53BP1, and RPA (Zhong *et al.* 1999; Paull *et al.* 2000; Wang *et al.* 2002; Fernandez-Capetillo *et al.* 2002; Lou *et al.* 2003; Stewart *et al.* 2003), may also be affected by these types of indirect effects. The indirect influences on Rad52 foci accumulation are echoed by the diverse roles of some of the genes identified (Alvaro *et al.* 2007), including transcription initiation (*IRC1*), clathrin-mediated vesicle trafficking (*IRC6*), degradation of cysteine (*IRC7*), and proteasome assembly (*IRC25*).

Analysis of the high-confidence DDS genes provides mechanistic insights into processes that generate and/or suppress the accumulation of DNA damage in unperturbed cells (Fig. 3h). High-confidence DDS genes include genes involved in DNA replication (*CSM3*, *CTF4*, *DBF4*, *DNA2*, *DPB4*, *MCM10*, *MRC1*, *ORC1*, *ORC2*, *POL1*, *POL12*, *POL2*, *POL3*, *POL31*, *POL32*, *PRI1*, *PRI2*, *RAD27*, *RTT105*, *RFA2*, and *TOF1*), DNA replication-associated histone disassembly and assembly (*ASF1*, *CAC2*, *HST3*, *POB3*, *RLF2*, and *RTT109*), sister chromatid cohesion (*CTF18*, *CTF8*, *CHL1*, *DCC1*, and *RAD61*), telomere homeostasis (*EST1*, *EST2*, *EST3*, and *STN1*), cell cycle control (*CLB5*, *CLN3*, *DBF2*, *SIC1*, and *SWI6*), and sumoylation, ubiquitination, and proteolysis (*CUZ1*, *DOA1*, *PRE4*, *RPN11*, *RPN4*, *RPT4*, *RPT6*, *SEM1*, *SLX5*, *SLX8*, *SMT3*, *ULP1*, and *UMP1*). In addition, genes involved in pathways known to repair and restart DNA replication forks were also identified, including homologous recombination (*MRE11*, *RAD50*, *XRS2*, *SAE2*, *RAD51*, *RAD52*, *RAD54*, *RAD55*, *RAD57*, *RAD59*, *SGS1*, *RMI1*, *TOP3*, *MMS4*, and *MUS81*), the Rtt101 cullin complex (*MMS1*, *MMS22*, *RTT101*, and *RTT107*), postreplication repair (*MMS2*, *RAD18*, *RAD5*, *RAD6*, and *UBC13*), and genes implicated in other repair pathways (*APN1*, *DIA2*, *ELG1*, *ESC2*, *IRC3*, *MLH1*, *MSH2*, *NSE1*, *RAD1*, *RNH202*, *RNH203*, *RRM3*, *SLX5*, *SLX8*, and *WSS1*). Genes encoding 2 components of the Rad17-Ddc1-Mec3 complex were also identified (*RAD17* and *DDC1*; Fig. 3h), indicating a role for the Mec1 DNA damage checkpoint in avoiding the accumulation of spontaneous DNA damage. Remarkably, the *RAD17* and *DDC1* genes were not identified in the Hug1 and Rnr3 expression screens; this is consistent with their roles in triggering the checkpoint required to derepress *HUG1* and *RNR3*. Mutations disrupting *HUG1* and *RNR3* repression (Basrai *et al.* 1999) were also identified in both the Hug1 and Rnr3 expression assays, including *rfx1Δ/crt1Δ*, *isw2Δ*, *itc1Δ*, *sin4Δ*, *hda1Δ*, and *hda3Δ*, but were generally not identified in the other DDR screens, consistent with these genes functioning in transcription repression and not functioning directly in the DDR. The high-confidence list also includes genes involved in transcription, including genes encoding subunits of the mediator (*SIN4* and *SRB5*), SAGA (*ADA2*, *NGG1*, and *SGF73*), Swr1 (*SWC4*, *VPS72*), COMPASS (*SWD1*), and Rpd3S/L (*RPD3*) complexes, and genes involved in transcriptional elongation (*DST1*, *SGV1*, and *SPT4*) and transcriptional silencing (*ESC1*, *ESC2*, and *SPT21*). The identification of transcriptional genes in both the high- and low-confidence lists is consistent with the possibility that transcriptional defects cause DNA damage, potentially by increasing collisions between the replication fork and the transcription machinery (Brambati *et al.* 2015). Taken together, these results indicate that DNA replication is a major source of spontaneous DNA damage in *S. cerevisiae* cells.

### Cell cycle analysis of DDR-inducing mutations is consistent with damage caused by DNA replication errors

**Fig. 4.**
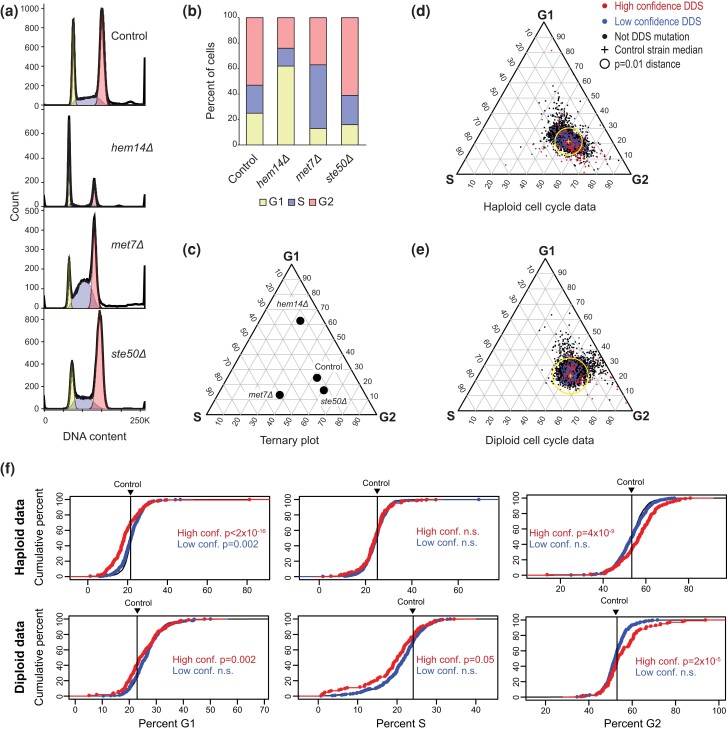
Cell cycle distributions for mutant strains with and without increased levels of DNA damage markers. a) Example FACS analyses of log-phase cultures of a haploid *S. cerevisiae* control strain and haploid strains with enriched G1 (*hem14Δ*), S (*met7Δ*), and G2 (*ste50Δ*) populations. FACS data (Koren *et al.* 2010) were plotted using FlowJo (FlowJo 2019) with cell count vs the measured SYBR-green area for each cell. Peaks for these haploid strains are at 1n and 2n DNA content. b) Stacked bar plots for the cell cycle distributions for the observations in panel a derived using FlowJo with the Dean–Jett–Fox model (Fox 1980) and fitted to account for cell–cell aggregation (see ‘Materials and methods’). c) Ternary plot of the cell cycle distributions of the observations in a). Samples only having G1, S, or G2 cells would be at the top, left, or right vertex, respectively. d) Ternary plot diagram for the cell cycle distribution of haploid strains colored by the DDR category (black = all, blue = low-confidence, and red = high-confidence). The cross is the position of the wild-type control strain. Strains outside the circle have significantly altered cell cycle distributions (*P* < 0.01). Inset shows the average deviation of all genes (black), high-confidence DDR genes (red), and low-confidence DDR genes (blue) relative to the geometric median of the control strain. e) Ternary plot diagram for diploid strains displayed as in d). f) The cumulative distribution of the percent of cells in the G1, S, and G2 phases for all mutations (black) and for the high- (red) and low- (blue) confidence DDR mutations. In almost all cases, the all-mutation and low-confidence-mutation curves overlap. Vertical lines indicate the percentage found in the control samples. *P*-values calculated by the Kolmogorov–Smirnov test with the distribution of all mutations as the null hypothesis; n.s., not significant.

A major function of DNA damage signaling is to trigger cell cycle checkpoints to prevent cell division in the presence of DNA damage (Blackford and Jackson 2017). We, therefore, reasoned that DDS gene mutations ought to perturb the timing of the cell cycle. To test this, we analyzed published FACS data for the *S. cerevisiae* haploid (Koren *et al.* 2010; Soifer and Barkai 2014) and diploid (Hoose *et al.* 2012) deletion collections to identify mutations that altered the percentage of cells in the G1, S, and G2 phases of the cell cycle during unperturbed growth (Supplementary Table 4). We first fitted each observation (Fig. 4a) so that the percentages of cells in the G1, S, and G2 phases summed to 100% (Fig. 4b). We then plotted each observation as a point on a ternary plot so that multiple observations could be combined and that cell cycle perturbations could be measured (Fig. 4c). The percentage of G1, S, and G2 cells was 21.5%, 25.1%, and 53.4% for the haploid control (*n* = 292) and 22.9%, 24.1%, and 53.0% for the diploid control (*n* = 219; Supplementary Fig. 5). Using these data, strains with cell cycle progression defects were identified (Fig. 4d and e; Supplementary Table 4).

DDS gene mutations as a group tended to perturb the cell cycle distribution of log-phase cultures. Comparison of the effects of all of the mutations tested with the effects of the mutations in high-confidence DDS genes revealed that the DDS mutations caused a general decrease in the percentage of cells in G1 phase (*P*-values of <2 × 10^−16^ and 0.002 for the haploid and diploid data, respectively; Kolmogorov–Smirnov test; Fig. 4f) and an increase in the percentage of cells in G2 phase (*P*-values of 4 × 10^−9^ and 2 × 10^−5^; Fig. 4f). This shift, along with a very minor or no effect on the percentage of S-phase cells, suggests that DDS gene defects cause an accumulation of cells with or very near to a 2N DNA content, which could be due to defects in the completion of DNA replication and/or triggering of the G2/M DNA damage checkpoint. In contrast, the distribution of the low-confidence DDS mutations was generally not different than the distribution of all mutations, most of which (88%) were not identified in any DDS screen (Fig. 4f). The results of this analysis are consistent with the idea that the accumulation of damage in many DDS gene mutants occurs during S phase and is consistent with the identification of high-confidence DDS genes that are associated with DNA replication.

### Genome-wide screen for deletion mutations causing increased GCR rates

In previous studies, we tested mutations in individual *S. cerevisiae* genes of interest and conducted a partial screen of the *S. cerevisiae* deletion collection (Chen and Kolodner 1999; Putnam, Hayes, *et al.* 2009; Chan and Kolodner 2011; Putnam *et al.* 2016; Nene *et al.* 2018; Srivatsan *et al.* 2019) and a screen of a set of mutations in an extensive subset of *S. cerevisiae* essential genes to identify mutations that cause increased GCR rates and identify genome instability-suppressing (GIS) genes. To facilitate comparisons between DDS genes and GIS genes, we performed a screen of the entire *S. cerevisiae* deletion collection for mutations causing increased GCR rates to expand our previous analyses to cover all nonessential genes. The strains recovered from the Hug1-EGFP cross also contained the duplication-mediated (dGCR) assay, which we included because increased dGCR rates can be detected using patch tests (Putnam, Hayes, *et al.* 2009). Haploid strains containing the dGCR assay and deletion mutations from the *S. cerevisiae* deletion collection were grown in 96-well plates, patched in triplicate using a Singer RoToR robot on YPD plates, and then replica plated onto GCR selection medium plates (Supplementary Fig. 6a). Our initial screen of patches identified 197 mutations affecting 193 genes as causing significantly increased number of papillae per patch (FDR < 0.05; Supplementary Fig. 6b). As expected, known mutations causing higher dGCR rates were more likely to be identified than those causing lower dGCR rates (Supplementary Fig. 6c). We then rescreened 620 strains, including the 197 strains with an FDR < 0.05, which resulted in identifying 52 candidate mutations that caused the largest increased patch scores, 24 of which were previously known to cause increased GCR rates (Supplementary Fig. 6d and e); this analysis defined 28 new candidate GIS genes that were incorporated into the analyses performed in this study (Supplementary Table 5). We performed a limited validation study in which mutant strains were reconstructed followed by measurement of GCR rates and found that 2 previously unidentified mutations causing the greatest number of papillae in the patch tests (*lip2Δ* and *mrm1Δ*) caused increased dGCR rates in reconstructed strains (Supplementary Table 6). It is unclear how these 2 defects cause increased GCR rates, as *LIP2* encodes an enzyme that adds lipoic acids to mitochondrial proteins, and *MRM1* encodes a mitochondrial RNA methylase. Combined with the results of previous studies, the number of known GCR-causing mutations from this and previous analyses is 419 (of 5,255 mutations tested), corresponding to 334 GCR-suppressing genes (Supplementary Table 5), many but not all of which have been extensively validated.

### Genome instability assays identify both general and assay-specific mutations

**Fig. 5.**
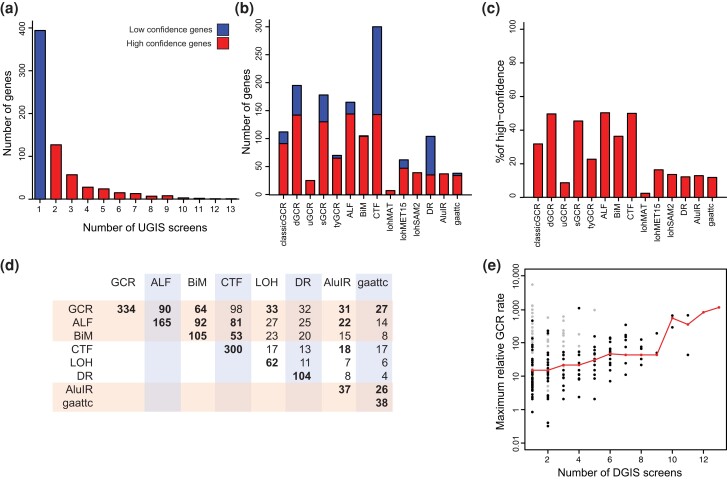
Comparison of DGIS screens. a) Histogram of the number of screens genes were identified in. High-confidence genes were identified in multiple screens. Low-confidence genes were identified in one screen. b) Total number of genes identified in each screen. c). Percentage of high-confidence DGIS genes identified in each screen, which was calculated by dividing the number of high-confidence genes identified in each screen b) by the total count of 286 high-confidence DGIS genes. d) Number of shared genes between assays. GCR, combination of the classicGCR, dGCR, uGCR, sGCR, and tyGCR assays. LOH, combination of the lohMAT, lohMET15, and lohSAM2 assays. e) Comparison of the maximum fold increased GCR rates of deletion mutants (black points) and hypomorphic mutants (gray points) with the number of screens that those alleles have been identified in. The line indicates the position of the median fold increased GCR rate for the alleles for each number of DGIS screens.

We combined data from studies using 14 genome instability assays to more comprehensively analyze genes thought to act to suppress genome instability. The assays used in these studies included (1) multiple GCR assays (Supplementary Table 5; Chen and Kolodner 1999; Putnam, Hayes, *et al.* 2009; Chan and Kolodner 2011; Putnam *et al.* 2016; Nene *et al.* 2018; Srivatsan *et al.* 2019), (2) an inverted Alu repeat instability assay (Zhang *et al.* 2013), (3) a GAA/TTC repeat instability assay (Zhang *et al.* 2012), (4) a direct repeat stability assay (Novarina *et al.* 2020), (5) chromosomal stability screens including the A-like faker (ALF) assay, the biallelic mating (BiM) assay, and the chromosome transmission failure (CTF) assay (Yuen *et al.* 2007; Stirling *et al.* 2011), and (6) screens for the loss of heterozygosity at the *MAT*, *MET15*, and *SAM2* loci (lohMAT, lohMET15, and lohSAM2; Andersen *et al.* 2008). Together, these screens evaluated 7,313 mutations and identified 286 high-confidence genes identified by multiple assays and 394 low-confidence genes identified by 1 assay (Fig. 5a; Supplementary Table 7). Remarkably, the overlap between the results of these screens is worse than that observed for the DDR screens (Fig. 5b–d), as no single screen identified more than 50% of the high-confidence genes. Commonly identified mutations, such as defects in DNA replication and homologous recombination, likely induce of damage that is misrepaired. Mutations restricted to one or a few assays likely reflect aspects of the different genetic readouts, such as the differences between assays identifying increased chromosomal rearrangements (GCR, DR, AluIR, and GAA/TTC stability assays), defects in chromosome transmission (ALF, BiM, and CTF assays), and increased sister chromosome recombination (ALF, BiM, and loh assays). Despite this, there is a trend in which mutations identified in more screens tend to cause greater increases in rates in GCR assays (Fig. 5e); note that some mutations, particularly hypomorphic mutations of essential genes, have been tested in a smaller number of assays and hence are more likely to be scored as low-confidence genes. Because of a broad range of genetic readouts in these assays, we call the identified genes the DGIS gene set.

### Mutations causing increased genome instability are not restricted to those causing increased DNA damage signaling

**Fig. 6.**
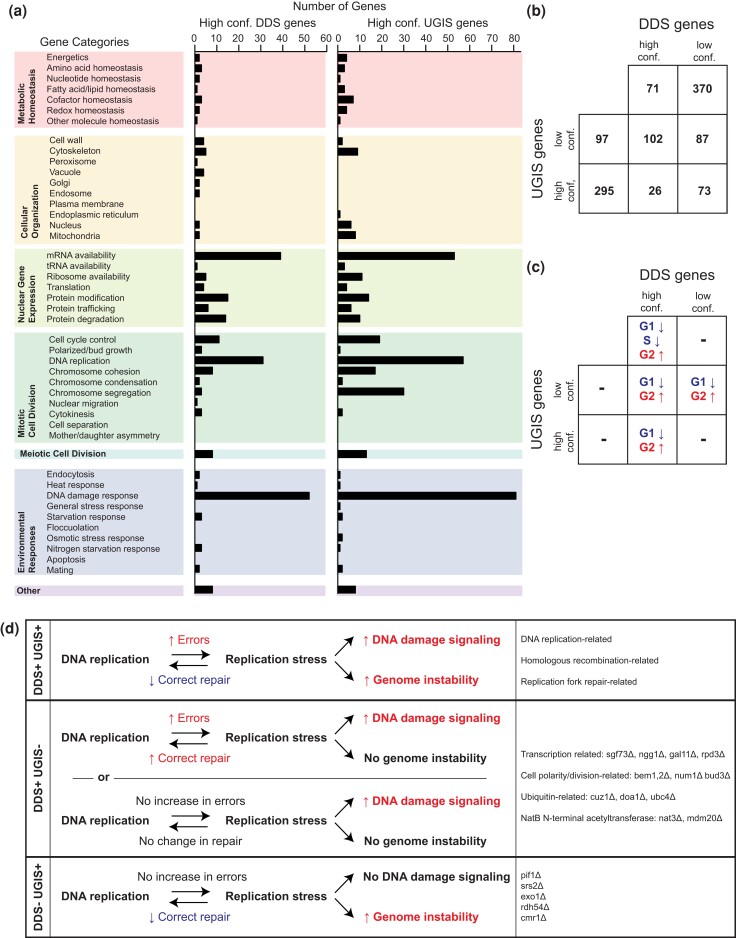
DDS and DGIS genes only partially overlap. a) Breakdown of the high-confidence DDS and DGIS genes into different functional categories shows that most categories are shared, with the exception that cell cycle control, chromosome cohesion, and chromosome segregation contain more genes in the high-confidence DGIS gene group than the high-confidence DDS gene group. b) Venn diagram of the high-confidence and low-confidence DDS and DGIS genes. c) Significant changes in the haploid log-phase cell cycle distribution for genes in different parts of the Venn diagram calculated as for Fig. 4f. d) Model for and examples of different categories of mutations.

Functional categorization of the high-confidence DDS and DGIS genes reveals that many are involved in common pathways, particularly DNA replication and the DNA damage response; however, the high-confidence DGIS genes also contain genes associated with cell cycle control, chromosome cohesion, and chromosome segregation (Fig. 6a). Direct comparison of the high- and low-confidence DDS and DGIS genes reveals that there are many genes identified in both types of assays (DDS+ DGIS+ genes), as well as genes identified in only one of the 2 types of assays (DDS+ DGIS− and DDS− DGIS+ genes; Fig. 6b; Supplementary Table 8). Consistent with the role of the DDR in arresting the cell cycle, the mutations with the strongest effects on the cell cycle (reducing the percentage of G1 cells and increasing the percentage of G2 cells) were mostly restricted to high-confidence DDS genes as was previously observed (Fig. 4f), whereas mutations in DDS− DGIS+ genes did not have a significant effect on cell cycle distributions (Fig. 6c).

DDS+ DGIS+ genes might be expected to suppress DNA damage and subsequent DNA damage signaling and genomic rearrangements (Fig. 6d). The genes/pathways identified by the DDS+ DGIS+ genes include the replication fork protection complex (*CSM3*, *MRC1*, and *TOF1*; Baretic *et al.* 2020), the Rtt101 cullin complex that regulates the stability of and repair events at stalled replication forks (*MMS1*, *MMS22*, *RTT101*, and *RTT107*; Duro *et al.* 2008; Vaisica *et al.* 2011; Buser *et al.* 2016), individual enzymes implicated in replication fork progression and repair events in the vicinity of the replication fork (*RRM3* and *WSS1*; Lopez-Mosqueda *et al.* 2016; Munoz-Galvan *et al.* 2017; Maddi *et al.* 2020), and pathways that promote replication fork reversal, including an Mph1-dependent and Smc5/6 regulated pathway (*MPH1* and genes encoding the Smc5-6 complex) and postreplication repair (*MMS2*, *RAD5*, *RAD6*, *RAD18*, and *UBC13*; Blastyak *et al.* 2007; Zheng *et al.* 2011; Xue *et al.* 2015). We also robustly identified genes involved in homologous recombination and DSB repair (*RAD50*, *MRE11*, *XRS2*, *RAD1*, *RAD10*, *RAD52*, *RAD51*, *RAD59*, *RAD54*, *RAD55*, *RAD57*, *TOP3*, *SGS1*, *RMI1*, *MMS4*, and *MUS81*) and genes involved in ribonucleotide excision repair (*RNH201*, *RNH202*, and *RNH203*). Several DNA repair-associated pathways were identified among the DDS+ DGIS+ pathways, including components of the DNA damage checkpoint (*RAD24*, *RAD17*, *DDC1*, *RAD9*, *TEL1*, and *RAD53*), protein sumoylation and sumo-targeted ubiquitination (*SMT3*, *ULP1*, *SLX5*, *SLX8*, *SIZ1*, and *ESC2*), and the nuclear pore (*NUP133*, *NUP60*, *NUP120*, *NUP145*, *NUP84*, and *NUP85*), which is known to play a role in HR.

The DDS+ DGIS− genes could either correspond to genes identified by mutations that result in increased damage where the damage is correctly repaired in most cases or to genes that prevent the DNA damage signaling components from recognizing normal DNA structures such as replication forks that do not correspond to DNA damage (Fig. 6d). These genes tend not to include DNA replication or repair genes but rather include genes involved in transcription (*ADA2*, *GAL11*, *GCR2*, *NGG1*, *RPD3*, *RPN4*, *SGF73*, *SIN4*, *SRB5*, *SUB1*, *SUM1*, *SWD1*, and *SWI4*), some aspects of ubiquitination (*CUZ1*, *DOA1*, and *UBC4*), genes associated with cell polarity, the actin cytoskeleton, and later steps during mitosis (*AIM44*, *BEM1*, *BEM2*, *BEM4*, *BUD3*, *DBF2*, *ECM33*, *ELM1*, *NUM1*, *SKT5*, *SLA1*, and *VRP1*), and the NatB *N*-terminal acetyltransferase (*MDM20* and *NAT3*). One intriguing possibility raised by this is that collisions between DNA replication forks and transcriptional machinery induce DNA damage that is efficiently repaired in most cases without generating genome rearrangements consistent with the weak effect that most R-loop causing mutations show in GCR assays (Gomez-Gonzalez *et al.* 2009; Wahba *et al.* 2011).

The DDS− DGIS+ genes likely promote the correct repair of DNA damage but do not normally suppress the formation of DNA damage. DDS− DGIS+ mutations include (1) *pif1* that causes defects in suppression of de novo telomere addition reactions at DNA damage; (2) different checkpoint mutations that disrupt the DDR and cell cycle delay/arrest in response to DNA damage (*chk1*, *ddc2*, *dun1*, *mec1*, *mec3*, and *mrc1-aq*) allowing aberrant repair to occur; (3) translesion synthesis, which may prevent the accumulation of DNA breaks during replication caused by an inability of the replicative DNA polymerases to extend past lesions or misincorporated bases (*rad30*, *rev1*, *rev3*, *rev7*, and *srs2*); and (4) mutations like *exo1* that by altering DSB resection, but not the overall efficiency of DNA repair, could change the balance between HR and pathways leading to misrepair.

## Discussion

Here, we investigated the relationship between the effect of mutations on inducing various DDR markers and inducing genome instability during unperturbed cell growth. Since these measurements have been made during unperturbed growth, the effects of the mutations observed are due to the accumulation and processing of endogenously generated DNA damage, which are most likely generated during DNA replication based on the analyses performed here. The results of 6 screens for mutations inducing DNA damage response markers, which employed 5 different types of assays, and from 14 screens for mutations that cause increased genome instability, which employed 6 different types of genome instability assays, identified 199 high-confidence DDS genes that were found in multiple screens, and similarly identified 286 high-confidence DGIS genes. Low-confidence genes may be due to (1) screening of a mutation in only a few assays, particularly for essential genes not present in the deletion collection, (2) false positive mutations, particularly for mutations that have not been extensively validated, and (3) mutations reflecting key differences between assay readouts, as some mutations are known to increase GCR formation in some GCR assays and not others and mutations involved primarily in chromosome transmission defects are unlikely to be identified in GCR assays.

It was our initial expectation that we would primarily identify genes in which mutations would both induce DNA damage response markers and genome instability because increased DNA damage would be likely to drive increased genome instability. Many such DDS+ DGIS+ genes were identified, and, based on their functional properties, these DDS+ DGIS+ genes are likely to act to (1) suppress DNA damage during replication, (2) promote repair of this DNA damage, and/or (3) prevent this DNA damage from being misrepaired. The DDS+ DGIS+ DNA repair pathways are consistent with at least 2 nonexclusive mechanisms by which cells process DNA replication defects. First, stalled replication forks may undergo fork reversal, which converts the 3-way replication fork junction to a 4-way junction in which the nascent strands are paired. This regressed fork contains a new double-stranded DNA end that can be extended by DNA polymerases, isomerized to rebuild a functional fork, or subject to homologous recombination with the template strand to rebuild a functional fork. Second, stalled or regressed replication forks may be subject to enzymatic cleavage to generate a double-stranded DNA break on 1 daughter duplex that can undergo homologous recombination with the sister chromatid, which is in proximity due to the cohesion of sister chromatids. In both mechanisms, replication fork-mediated and cohesin-mediated coordination of the damaged and undamaged duplexes would be expected to both promote repair and help channel the damage away from misrepair events (for example nonallelic homologous recombination or targeting of the double-stranded end in the regressed fork by the nonhomologous end joining or de novo telomere addition pathways). These misrepair events would give rise to genome rearrangements observable by one or more of the genome instability assays analyzed here. Repair-associated pathways, such as the DNA damage checkpoint, sumoylation by Mms21, SUMO-targeted ubiquitin ligation by the nuclear pore-associated Slx5-Slx8 complex, and protein degradation, have been implicated in the regulation of components of the fork repair processes outlined above (Argunhan *et al.* 2021; Cappadocia *et al.* 2021; Dhingra and Zhao 2021).

The identification of additional categories of genes suppressing DNA damage and genome instability (e.g. DDS− DGIS+ and DDS+ DGIS−) besides the DDS+ DGIS+ category was not initially expected but has important biological implications. An important difference between these types of assays is that cytological assays for DDR markers measure responses in the bulk of the cells (e.g. the “averaged” response), whereas genome instability assays detect increased levels of rare events, which are generally not observable in the absence of genetic selection. The DDS− DGIS+ genes could reflect genes in which mutations result in the misrepair of normal levels of spontaneous DNA damage that is not detected as induction of DNA damage or result in the inappropriate recognition of normal DNA as damage. The DDS+ DGIS− genes likely reflect genes in which mutations cause increased DNA damage where the DNA damage is correctly repaired such that genome instability does not occur. Consistent with the idea that the DDS and DGIS gene categories define mechanistically distinct groups of genes, DDS− DGIS+ and DDS+ DGIS+ mutations are functionally distinct in mutation accumulation experiments, where multiple cultures are grown in parallel and analyzed after many generations of growth. In these experiments, *mre11Δ* and *rad27Δ* strains (DDS+ DGIS+) accumulated large numbers of rearrangements, whereas *pif1Δ* strains (DDS− DGIS+) did not (Serero *et al.* 2014), despite having similar GCR rates (Chen and Kolodner 1999). These differences not only affect how they can be studied in model organisms but also have implications as to how these defects can be therapeutically targeted in human cancers. An important implication of the existence of different classes of DDS and DGIS genes and the imperfect overlap of DDR markers with genome instability suggests caution in inferring the presence or lack of increased genome instability based on the presence or lack of markers for increased DNA damage.

## Supplementary Material

jkae064_Supplementary_Data

## Data Availability

All data, yeast strains, and plasmids underlying this article will be shared on request to the corresponding author. Supplementary Tables 1–3 contain the Hug1-EGFP measurements, the Ddc2-EGFP foci measurements, and the compiled information for the high- and low-confidence DDS genes. Supplementary Table 4 contains the cell cycle distribution data for the mutant strains. Supplementary Tables 5–7 contain the list of GCR-suppressing genes, GCR rates, and the high- and low-confidence genome instability-suppressing genes. Supplementary Table 8 contains the merged information for all mutations. Supplemental material available at G3 online.
