# Supplementary material for: Identification of different classes of genome instability suppressor genes through analysis of DNA damage response markers
Source: G3 (Bethesda). 2024 Mar 25;14(6):jkae064. doi: 10.1093/g3journal/jkae064 (PMC11152081; doi:10.1093/g3journal/jkae064)

A

RDKY8174

MATα
lyp1::TRP1
cyh2-Q38K
iYFR016C::P_{MFA1}*-LEU2*
HUG1-EGFP.hphNT1
can1::P_{LEU2}*-NAT*
yel072w::CAN1-URA3

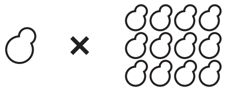

BY4741 deletion collection

*MAT***a**
yfgΔ::kanMX4

SGA Step	Cross	Diploid Selection (x2)	Presporulation	Sporulation	Diploid Killing (x2)	Haploid Selection (x2)
**Medium**	YPD	YPD<br>+hygromycin<br>+G418	PreSpo	Spo	CSM-Lys-Leu-Ura<br>+thialysine<br>+cycloheximide<br>+hygromycin<br>+nourseothricin<br>+G418	CSM-Leu-Ura<br>+hygromycin<br>+nourseothricin<br>+G418
**Selected Genotype**<br><br>**and**<br><br>**Markers under Selection**		*MAT***a***/MATα*<br>*LYP1/lyp1::TRP1*<br>*CYH2/cyh2-Q38K*<br>*iYFR016C/iYFR016C::*P_{MFA1}*-LEU2*<br>*HUG1/HUG1-EGFP.hphNT1*<br>*CAN1/can1::*P_{LEU2}*-NAT*<br>*YEL072W/yel072w::CAN1-URA3*<br>*YFG/yfgΔ::kanMX4*			*MAT***a**<br>*lyp1::TRP1*<br>*cyh2-Q38K*<br>*YFR016C::*P_{MFA1}*-LEU2*<br>*HUG1-EGFP.hphNT1*<br>*can1::*P_{LEU2}*-NAT*<br>*yel072w::CAN1-URA3*<br>*yfgΔ::kanMX4*	*MAT***a**<br>*lyp1::TRP1*<br>*cyh2-Q38K*<br>*YFR016C::*P_{MFA1}*-LEU2*<br>*HUG1-EGFP.hphNT1*<br>*can1::*P_{LEU2}*-NAT*<br>*yel072w::CAN1-URA3*<br>*yfgΔ::kanMX4*

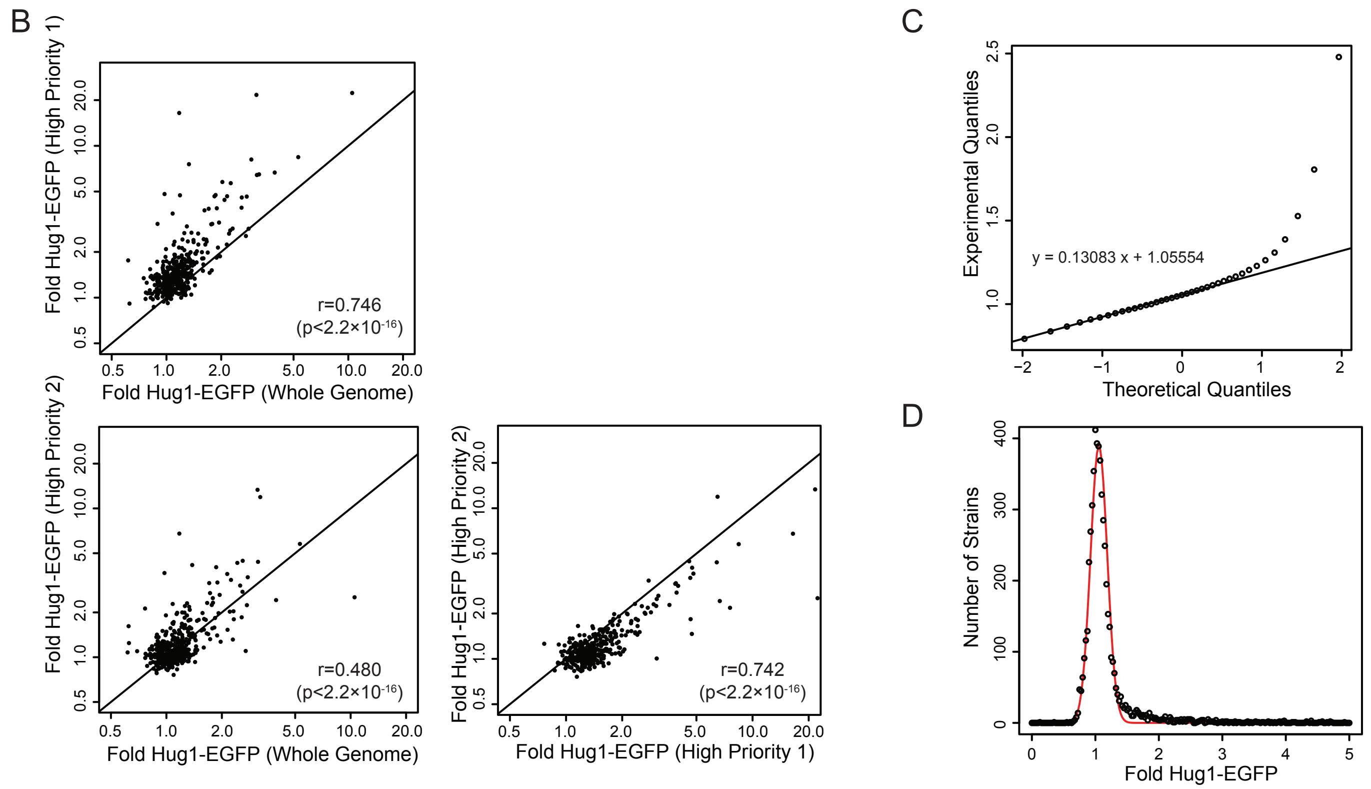

Supplement: jkae064_Supplementary_Data [file jkae064_supplementary_data.zip › Supplementary_Figure_3_G3-2024-404884.pdf]
