# Supplementary material for: Identification of different classes of genome instability suppressor genes through analysis of DNA damage response markers
Source: G3 (Bethesda). 2024 Mar 25;14(6):jkae064. doi: 10.1093/g3journal/jkae064 (PMC11152081; doi:10.1093/g3journal/jkae064)
Supplement: jkae064_Supplementary_Data [file jkae064_supplementary_data.zip › Supplementary_Figure_4_G3-2024-404884.pdf]

A

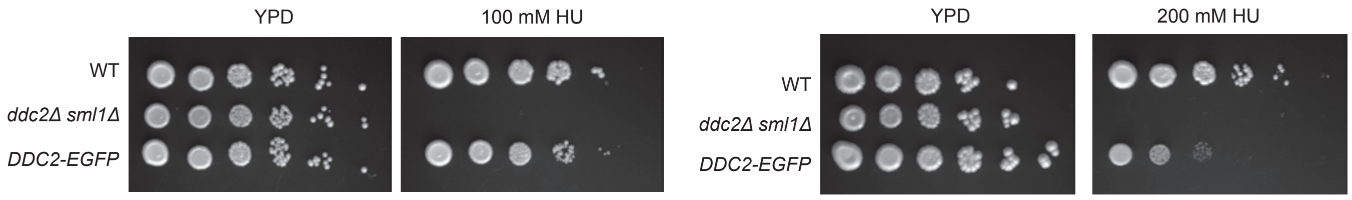

B

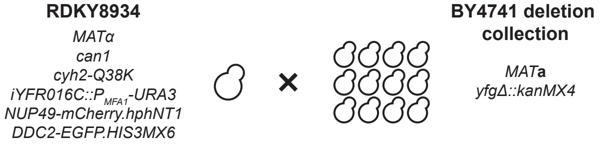

SGA Step	Cross	Diploid Selection (x2)	Presporulation	Sporulation	Diploid Killing (x2)	Haploid Selection (x2)
**Medium**	YPD	YPD<br>+hygromycin<br>+G418	PreSpo	Spo	CSM-Arg-His-Ura<br>+canavanine<br>+cycloheximide<br>+hygromycin<br>+G418	CSM-His-Ura<br>+hygromycin<br>+G418
**Selected Genotype**<br><br>**and**<br><br>**Markers under Selection**		*MAT**a**/MATα*<br>*CAN1/can1*<br>*CYH2/cyh2-Q38K*<br>*iYFR016C/iYFR016C::P_{MFA1}-URA3*<br>*NUP49/NUP49-mCherry.hphNT1*<br>*DDC2/DDC2-EGFP.HIS3MX6*<br>*YFG/yfgΔ::kanMX4*			*MAT**a***<br>*can1*<br>*cyh2-Q38K*<br>*iYFR016C::P_{MFA1}-URA3*<br>*NUP49-mCherry.hphNT1*<br>*DDC2-EGFP.HIS3MX6*<br>*yfgΔ::kanMX4*	*MAT**a***<br>*can1*<br>*cyh2-Q38K*<br>*iYFR016C::P_{MFA1}-URA3*<br>*NUP49-mCherry.hphNT1*<br>*DDC2-EGFP.HIS3MX6*<br>*yfgΔ::kanMX4*
