# Supplementary material for: Identification of different classes of genome instability suppressor genes through analysis of DNA damage response markers
Source: G3 (Bethesda). 2024 Mar 25;14(6):jkae064. doi: 10.1093/g3journal/jkae064 (PMC11152081; doi:10.1093/g3journal/jkae064)
Supplement: jkae064_Supplementary_Data [file jkae064_supplementary_data.zip › Supplementary_Methods_G3-2024-404884.docx]

**SUPPLEMENTARY MATERIAL FOR:**

Identification of different classes of genome instability suppressor genes through analysis of DNA damage response markers

By

Bin-Zhong Li^1^, Richard D. Kolodner^1, 2, 3, 4^ and Christopher D. Putnam^1, 5^

From

Ludwig Institute for Cancer Research^1^, Department of Cellular and Molecular Medicine^2^, Moores-UCSD Cancer Center^3^, Institute of Genomic Medicine^4^, and Department of Medicine^5^, University of California San Diego School of Medicine, 9500 Gilman Drive, La Jolla, CA 92093-0669

Address correspondence to:

Christopher D. Putnam

cdputnam@health.ucsd.edu

(858) 534-5125 (phone)

(858) 534-7750 (fax)

**Supplementary Methods**

**Bait strain construction**

The bait strain containing the *HUG1-EGFP* marker was created by integrating a *EGFP-hphNT1* cassette amplified by PCR from pYM25 (EUROSCARF P30237) using the primers 5’-CCG TCG AAC GTC GCG GCG GTC TTT CTG ACA TTG GTA AGA ATA CTT CCC AAC cgtacgctgcaggtcgac-3’ and 5’-TTG TTC TTT TCC TAT CAT TGG CCT ACA AAA AAA AAG AGA AGC ATG CTC TTA atcgatgaattcgagtcg-3’ onto the 3’ end of the *HUG1* coding sequencing in the strain RDKY7635 (Putnam et al. 2016) to create RDKY8174 (*MATα* *his3Δ200 hom3-10 leu2Δ0 trp1Δ63 ura3Δ0 lyp1::TRP1 cyh2-Q38K iYFR016C::P_MFA1_-LEU2 can1::P_LEU2_-NAT yel072w::CAN1-URA3 HUG1-EGFP.hphNT1*); note that this strain contains the dGCR assay markers.

The bait strain containing the *DDC2-EGFP* marker was created by: [1] crossing RDKY7597 (*MAT***a** *his3Δ200 hom3-10 leu2Δ0 lys2-10A trp1Δ63 ura3Δ0 iYFR016C::P_MFA1_-LEU2*) with RDKY7629 (*MATα* *his3Δ200 hom3-10 leu2Δ0 trp1Δ63 ura3Δ0 lyp1::TRP1 cyh2-Q38K iYFR016C::P_MFA1_-LEU2 can1::P_LEU2_-NAT* ;(Putnam et al. 2016)), [2] replacing the *iYFL016C::P_MFA1_-LEU2* with *iYFL016C::P_MFA1_-URA3* in a *MAT***a** haploid spore clone, [3] re-crossing the resulting strain to a *MATα* haploid spore clone from the same RDKY7597 x RDKY7629 cross, [4] screening for a *MATα* haploid spore clone with the *iYFL016C::P_MFA1_-URA3* marker, [5] selecting for a mutation in *CAN1* by plating on CSM-Arg plates containing 60 μg/mL canavanine (Sigma Aldrich), [6] integrating an *mCherry-hphNT1* fragment made by PCR using pBS35 as templete (Addgene #83797) with the primers 5’- GAA TCG CCG TGT TAC ATC AAA AAA CGA AAA CAC TGG CAT CAT TGA GCA TA cgtacgctgcaggtcgac-3’ and 5’- CAT TTG TAC TTG TTA TAC GCA CTA TAT AAA CTT TCA GGG CGA TTT ACT CA atcgatgaattcgagctcg-3’ at the C-terminus of *NUP49*, and [7] integrating a PCR product containing the *EGFP-HIS3* fragment amplified from pFA6a-GFP(S65T)-HIS3MX6 (Addgene #41598) with the primers 5’- ATC TAA CCA CAC TAG AGG AGG CCG ATT CAT TAT ATA TCT CAA TGG GAC TG tcgtacgctgcaggtcga-3’ and 5’-ACA AGG TTT CTA TAA AGC GTT GAC ATT TTC CCC TTT TGA TTG TTG CCT TA atcgatgaattcgagctcg-3’ at the C-terminus of *DDC2*. The resulting strain was RDKY8934 (*MATα* *his3Δ200 hom3-10 leu2Δ0 trp1Δ63 ura3Δ0 can1 cyh2-Q38K iYFR016C:P_MFA1_-URA3 NUP49-mCherry.hphNT1 DDC2-EGFP.HIS3MX6*).

**Systematic genetic crosses**

The *HUG1-EGFP* containing bait strain RDKY8174 was crossed twice to a selected set of the *S. cerevisiae* deletion collection (BY4741 strains, *MAT***a** *his3Δ1 leu2Δ0 met15Δ0 ura3Δ0*) and once to the entire collection of BY4741 *MAT***a** strains using a RoToR pinning robot (Singer Instruments). The *DDC2-EGFP.HIS3MX6* containing bait strain was crossed to a set of 468 BY4741 *MAT***a** deletion strains containing mutations selected as causing increased Hug1-EGFP expression or increased GCR rates or the *leu2Δ::kanMX4* control mutation.

For crossing the *HUG1-EGFP* containing bait strain to the deletion collection, the bait strain was grown on YPD and crossed in quadruplicate with arrayed deletion strains by pinning onto fresh YPD agar plates using a Singer RoToR followed by growth for two days. Cells were subjected to two rounds of pinning onto diploid selection medium (YPD-agar containing 200 μg/mL geneticin (G418, ThermoFisher) and 200 μg/mL hygromycin (Gibco)) and grown for 1-2 days at 30˚C. The cells were then pinned onto pre-sporulation medium (15 g Difco nutrient broth (BD Biosciences), 5 g Bacto-yeast extract (ThermoFisher), 10 g Bacto-agar (ThermoFisher), and 62.5 mL 40% glucose (ThermoFisher) per 500 mL) and grown for 3 days at 30˚C. Cells from the pre-sporulation medium were then pinned onto sporulation medium (10 g potassium acetate, 0.05 g zinc acetate, 20 g Bacto-agar per liter, containing a final concentration of 50 μg/mL G418 and 50 μg/mL hygromycin) and incubated for 7 days at 30˚C. The resulting spore-containing cells were then subjected to two rounds of pinning onto diploid killing medium (1.7 g yeast nitrogen base without amino acids and without ammonium sulfate (BD Biosciences), 1 g L-glutamic acid monosodium salt, 2 g CSM dropout mix without lysine, leucine, and uracil (US Biological), 20 g Bacto-agar, 50 mL of 40% glucose per liter, containing a final concentration of 50 μg/mL thialysine (Sigma Aldrich), 10 μg/mL cycloheximide (Sigma Aldrich), 200 μg/mL G418, 200 μg/mL hygromycin, and 100 μg/mL nourseothricin) followed by growth for 5 days at 30˚C for the first pinning and 2 days at 30˚C for the second pinning. Cells were then subjected to two rounds of pinning followed by growth for 2 days at 30˚C on haploid selection medium (1.7 g yeast nitrogen base without amino acids and without ammonium sulfate, 1 g L-glutamic acid monosodium salt, 2 g CSM dropout mix without leucine and uracil, 20 g Bacto-agar, 50 mL of 40% glucose per liter, containing a final concentration of 200 μg/mL G418, 200 μg/mL hygromycin, and 100 μg/mL nourseothricin). Then the cells were pinned and grown on YPD-agar followed by storage at -80˚C in YPD media containing 20% (v/v) glycerol.

Crossing the *DDC2-EGFP.HIS3MX6* containing bait strain was performed by essentially the same procedure, except for the use of different compositions of diploid killing medium (1.7 g yeast nitrogen base without amino acids and without ammonium sulfate, 1 g L-glutamic acid monosodium salt, 2 g CSM dropout mix without arginine, histidine, and uracil, 20 g Bacto-agar, 50 mL of 40% glucose per liter, containing a final concentration of 50 μg/mL canavanine, 10 μg/mL cycloheximide, 200 μg/mL G418, and 200 μg/mL hygromycin) and haploid selection medium (1.7 g yeast nitrogen base without amino acids and without ammonium sulfate, 1 g L-glutamic acid monosodium salt, 2 g CSM dropout mix without uracil and histidine, 20 g Bacto-agar, 50 mL of 40% glucose per liter, containing a final concentration of 200 μg/mL G418 and 200 μg/mL hygromycin).

**Cell cycle distribution data analysis**

Data compilation. The cell cycle distributions of log phase cells for the *MAT***a** BY4741 haploid deletion collection and the homozygous diploid BY4743 deletion collection were previously published by others (Hoose et al. 2012; Koren et al. 2010; Soifer and Barkai 2014). For the diploid deletion collection data, the published supplement containing cell cycle parameters from the Dean-Jett-Fox model (Fox 1980) as implemented in FlowJo were used (Hoose et al. 2012). For the haploid deletion collection data, FACS data were downloaded from the FlowRepository (Spidlen et al. 2012) and were analyzed using the Denn-Jett-Fox model in FlowJo version 10 (FlowJo 2019) after removing doublets that ran perpendicular to the laser by gating on a forward scatter height (FSC-H) vs. forward scatter width (FSC-A) plot. Note that cell-cell doublets that are parallel to the laser cannot be excluded by this gate. Cell cycle distribution data were filtered for problematic observations. These observations included those flagged by the original authors (Hoose et al. 2012), those with problematic fits based on visual inspection, those with very small numbers of cells, or those with abnormal ploidies. All observations, including those excluded from downstream analyses, are reported in Supplementary Table 4.

Determination of aggregation-corrected cell-cycle distributions. Ternary plots are ideal ways to analyze data in which three variables sum to a constant value; in the case of cell cycle data, the percent of G1, S, and G2 cells (ignoring the sub-G1 and super-G2 populations) can be divided by their sum so that the sum is scaled to 1. This simple scaling, however, ignores the information in the super-G2 population about cell-cell aggregation present in the sample, which would tend to systematically decrease the overall levels of the G1 and S populations; G1-G1 aggregates will be scored with the G2 population, and aggregates containing S and G2 cells will be scored with the super-G2 population.

To take aggregation into account, distributions with a super-G2 population were fitted with a model in which cell-cell aggregates were assumed to be cell-cycle independent and aggregates larger than two cells were ignored, as they were assumed to be present at vanishingly small amounts in the sample. In this model, the underlying fractions of cells in the G1, S, and G2 populations (*f_G1_*, *f_S_*, *f_G2_*, where *f_G1_*+*f_S_*+*f_G2_*=1) and the fraction of cells in aggregates (0≤*f_A_*≤1) were determined by fitting the following equations:

Observed percentage G1 = *k* ( 1 – *f_A_* ) *f_G1_*

Observed percentage S = *k* ( 1 – *f_A_* ) *f_S_*

Observed percentage G2 = *k* [ ( 1 – *f_A_* ) *f_G2_* + *f_A_ f_G1_*^2^ ]

Observed percentage super-G2 = *k f_A_* *(* 2 *f_G1_f_S_* + 2 *f_G1_f_G2_* + *f_S_*^2^ + 2 *f_S_f_G2_* + *f_G2_*^2^ )

where *k* is the sum of the observed percentages of G1, S, G2, and super-G2 cells. These four equations have only three unknowns (*f_G1_*, *f_S_*, *f_A_*), and the parameters were determined using Powell minimization as implemented in the SciPy python package (Virtanen et al. 2020). In general, the fitted values of *f_G1_*, *f_S_*, and *f_G2_* were only modestly different from those calculated by scaling the sum of the G1, S, and G2 populations to 1, even in the presence of moderate fractions of aggregation. Downstream analyses used the aggregation-corrected frequencies *f_G1_*, *f_S_*, and *f_G2_*.

Projecting cell cycle data onto a ternary plot. With each observation expressed as the fractional distribution of cells in each cell cycle phase (*f_G1_*, *f_S_*, *f_G2_*, where *f_G1_*+*f_S_*+*f_G2_*=1), each observation was represented as a two-dimensional point on a ternary plot. With G1 at top vertex of the graph (0.5, √3/2), S at left vertex (0, 0), and G2 at the right vertex (0, 1), the fractional distribution can be related with a point (*x*, *y*) on the ternary plot using the following equations:

*x* = 0.5 [ ( 2*f_G2_* + *f_G1_* ) / ( *f_G1_* + *f_S_* + *f_G2_* ) ]

*y* = ( √3 / 2 ) [ *f_G1_* / ( *f_G1_* + *f_S_* + *f_G2_* ) ]

Merging multiple observations. To merge multiple observations together, the geometric median of all projected observations in the ternary plot was determined using Weiszfeld’s algorithm, which iteratively re-weights points to minimize the weighted sum of squares of the distances of the points with the median (Weiszfeld 1937). This merging procedure was performed on multiple observations of a mutation, but in principle could be performed on all observations of all mutations affecting a gene, a complex, or a pathway. The resulting median in the ternary plot (*x_m_*, *y_m_*) was converted back to a fractional distribution (*f_G1_*, *f_S_*, *f_G2_*) using the relationships:

*f_G1_* = 2 *y_m_* / √3

*f_G2_* = ( 2 *x_m_* – *f_G1_* ) / 2

*f_S_* = 1 – *f_G1_* – *f_G2_*

Identification of mutations causing altered cell-cycle distributions. First, a null model was generated using the ternary plot distances between the geometric median of the control strain observations (Supplementary Fig. 5A) and each individual experimental (mutant) observation. These distances had a non-normal distribution, with reduced observations at low distances and a distribution skewed to longer distances (Supplementary Fig. 5B). Because these ternary plot distances are two dimensional, we compared the control distances to a theoretical distribution of distances arising from points in which the *x* and *y* values were normally distributed about the origin with a standard deviation of 1 (Supplementary Fig. 5B). We term this error model *the two independent Gaussian error model*. A linear relationship in the quantile-quantile plot at short quantiles suggests that the observed distributions fit this theoretical model with a subset of outliers at large values. From the quantile-quantile plot, the standard deviation for the control distances in haploid data was 0.0263 and the diploid data was 0.0340 (Supplementary Fig. 5C).

Next, all observations for each deletion allele were compared to the null models (Supplementary Fig. 5D,F). Haploid strain observations were compared to the haploid null model, and diploid strain observations were compared to the diploid null model. For a deletion allele with *n* observations, the deviation vector for each observation *i, Δ****p_i_***, was calculated:

*Δ****p_i_*** = ( *Δx_i_*, *Δy_i_* ) = ( *x_i_* – *x_c_*, *y_i_* – *y_c_* )

where *x_i_* and *y_i_* are the ternary plot *x* and *y* values for the observation and *x_c_* and *y_c_* correspond to the *x* and *y* values of the geometric median of the control observations. All of the observed deviation vectors for the deletion allele were then summed, and the length of the summed vector, |*Δ****p***| was determined:

|*Δ****p***| = [ Σ(*Δx_i_*)^2^ + Σ(*Δy_i_*)^2^] ^1/2^

The observed length was then compared to a million simulations in which *n* deviation vectors were randomly generated, and the length of the sum of these randomly generated vectors was determined. For the generation of each simulated observation, the length of the vector was taken from the null model and the orientation was randomly assigned. The *p*-value for the change in the cell-cycle distribution was taken as the fraction of the million simulations that had summed deviation vectors with longer lengths than the observed length of the summed deviation vectors. The Benjamini-Hochberg false-discovery rate correction for multiple tests was then applied to the resulting *p*-values for all of the deletion alleles (Supplementary Fig. 5E,G; (Benjamini and Hochberg 1995)).

This analysis identified 939 mutations in 911 genes in either or both the haploid and diploid datasets with a significantly altered cell cycle distributions. These samples had an FDR of less than 0.01 and a ternary plot distance that was 3 or more times the standard deviation, which corresponds to p<0.01 in the two independent Gaussian error model described above. Increased fractions of G1 phase cells were caused by many defects affecting translation, including mutations in genes encoding ribosomal subunits, ribosomal RNA transcription, and ribosome assembly (Supplementary Table 4), which is consistent with previous observations (Hoose et al. 2012; Moore 1988; Popolo et al. 1982; Soifer and Barkai 2014). In addition, mutations affecting mitochondrial function, adenine biosynthesis, NAD biosynthesis, and vacuole acidification also resulted in increased fractions of G1 phase cells (Supplementary Table 4). Increased fractions of S phase cells were observed for mutations affecting cell cycle progression, nucleotide biosynthesis, DNA replication, and histone gene expression (Supplementary Table 4). Increased fractions of G2 phase cells were observed for mutations affecting several DNA repair pathways, including HR, post-replication repair, the Mre11-Rad50-Xrs2 complex, the Sgs1-Top3-Rmi1 complex, and the Mms1-Mms22-Rtt101-Rtt107 cullin complex (Supplementary Table 4). In addition to DNA repair defects, defects in pathways involved in mitosis also increased the fraction of G2 phase cells, including mutations affecting cell cycle progression, chromosome cohesion and segregation, the actin cytoskeleton, and cytokinesis (Supplementary Table 4).

**Supplementary Figure Legends**

**Supplementary Figure 1. DDR marker pathways *in S. cerevisiae*.** DNA damage checkpoint is triggered by the recognition of DNA damage through Tel1 or Mec1-Ddc2, which activates these protein kinases to phosphorylated Rad53 and its coactivators Rad9 and/or Mrc1. Rad53 then phosphorylates Dun1 and Mck1 (Ciccia and Elledge 2010; Lanz et al. 2019; Li et al. 2019; Putnam et al. 2009). The checkpoint pathway also impinges on ribonucleotide reductase (RNR) regulation at several steps. **(1)** Phosphorylation of Ixr1 to induce *RNR1* expression (Tsaponina et al. 2011). **(2)** Phosphorylation of the Rnr1 inhibitor Sml1, leading to its ubiquitination and degradation (Chabes et al. 1999; Zhang et al. 2007; Zhao et al. 2001; Zhao et al. 1998). **(3)** Induction of *RNR2*, *RNR3*, and *RNR4* by phosphorylation of Rfx1/Crt1 and alleviation of its repression by the Tup1/Ssn6 general suppressors (Huang et al. 1998). **(4)** Induction of HUG1, which encodes an Rnr2-Rnr4 repressor, also by phosphorylation of Rfx1/Crt1 (Basrai et al. 1999; Meurisse et al. 2014); simultaneous induction of Hug1 expression along with the expression of RNR subunits is thought to modulate RNR activity. **(5)** Release of Rnr2-Rnr4 from sequestration in the nucleus through phosphorylation of the sequestering protein Wtm1, which disrupts the interaction with Rnr2-Rnr4, and degradation of Dif1, which helps import Rnr2-Rnr4 into the nucleus (Lee and Elledge 2006; Lee et al. 2008; Wu and Huang 2008). Many of these responses have been used experimentally to monitor activation of the DNA damage checkpoint.

**Supplementary Figure 2. Selection of Hug1-EGFP DDR marker expression for analysis of mutant strains. A.** We fused EGFP to the C-terminus of four genes whose expression was substantially increased (*DDR2*, *HUG1*, and *RNR3*) or decreased (*DSE2*) by induction of DNA damage due to telomere deprotection (Greenall et al. 2008). Strains containing one of the four fusions were grown to mid-log phase and treated with and without 100 mM HU for 2 hours to induce DDR activation. The EGFP signal was measured by FACS. The levels of Rnr3-EGFP and Hug1-EGFP were substantially up-regulated by HU treatment, consistent with previous studies (Basrai et al. 1999; Huang et al. 1998), but Dse2-EGFP and Ddr2-EGFP levels were not substantially changed. **B.** Wildtype, *pph3Δ*, *rad53Δ* *sml1Δ*, and *mec1Δ* *sml1Δ* cells containing the *HUG1-EGFP* marker were grown overnight in YPD, diluted 10-fold into YPD + 200 mM HU for 240 minutes, pelleted and then resuspended into fresh media lacking HU. Hug1-EGFP expression was measured using FACS. Hug1-EGFP levels were rapidly induced by HU in the wild-type and *pph3Δ* cells as measured by the mean EGFP-A (EGFP area) value of all measured cells and began to decrease after 30 minutes in media lacking HU. The induction in *pph3Δ* cells was somewhat greater than wildtype cells, consistent with a role of Pph3 in dephosphorylating targets of the DNA damage checkpoints. In contrast, no induction was observed in *meclΔ sml1Δ* or *rad53Δ sml1Δ* strains, consistent with the known requirement of the Mec1 and Rad53 kinases upstream of the transcriptional activation of the *HUG1* gene (Basrai et al. 1999). **C.** To test if the *HUG1-EGFP* marker was functional, we tested the effect of combining the *HUG1-EGFP* allele with *dun1Δ*, as loss of *HUG1* has been reported to suppress the hydroxyurea sensitivity of the *dun1Δ* mutation (Basrai et al. 1999). Both the *dun1Δ hug1Δ* double mutant and the *dun1Δ HUG1-EGFP* double mutants were more resistant to hydroxyurea, indicating that the Hug1-EGFP fusion construct was not functional; however, use of the *HUG1-EGFP* allele as a marker of DDR activation depends on the function of the *HUG1* promoter, not the function of the Hug1 protein. Consistent with this, previous studies have similarly used a reporter in which the *HUG1* coding sequence was deleted and replaced by GFP or firefly luciferase (Ainsworth et al. 2012; Benton et al. 2007). These studies demonstrated that expression driven by the *HUG1* promoter is responsive to a wide variety of DNA damaging agents, but not other types of cellular stress.

**Supplementary Figure 3. Systematic generation and analysis of *HUG1-EFP* strains.** **A.** Scheme for systematic generation of *HUG1-EGFP* strains by SGA (see Methods for details) that shows the media used at each step and the markers under selection at each step (highlighted in red). **B.** Independent Hug1-EGFP expression measurements are highly correlated. Subsets of the deletion collection were independently crossed with the *HUG1-EGFP* query strain and Hug1-EGFP expression in haploid progeny was measured by FACS. The data from three independent experiments was analyzed: “High Priority 1” (412 valid measurements), “High Priority 2” (482 valid measurements), and “Whole Genome” (4,936 valid measurements). The High Priority subset of mutants that was crossed twice was a set of predicted genome-instability causing mutations based on previous bioinformatic and experimental analyses (Putnam et al. 2012). Plots of the fold increase in Hug1-GFP expression for strains with the same genotypes from each experiment show that the observed values were highly correlated; Pearson correlation coefficient (*r*) and *p*-value for the null hypothesis that the true correlation is zero are shown for each pairwise comparison. **C.** Fold increases in Hug1-EGFP expression levels are normally distributed as shown by the linear relationship in the quantile-quantile plot that compares the quantiles of the experimental fold increase in Hug1-EGFP expression levels against the quantiles of a theoretical normal distribution. Outliers at higher quantiles correspond to strains with high levels of Hug1-EGFP expression. Regression of linear portion of the plot indicates that a fitted normal distribution of the data has a mean fold Hug1-EGFP of 1.05 and a deviation of 0.13. **D.** Histogram of all measured Hug1-EGFP expression levels relative to the control strain (closed circles) fitted to a Gaussian distribution (red curve) described by the parameters from the fit of the quantile-quantile plot (panel C).

**Supplementary Figure 4. Systematic generation and analysis of *DDC2-EGFP* strains.** **A.** The Ddc2-EGFP fusion protein is functional, as the *DDC2-EGFP* strain is viable in the absence of the *sml1Δ* mutation, required for the viability of the *ddc2Δ* mutation (Rouse and Jackson 2000). In addition, the strain containing the Ddc2-EGFP fusion is also more resistant to hydroxyurea than the *ddc2Δ* *sml1Δ* double mutant deletion. **B.** Scheme for systematic generation of *DDC2-EGFP* strains by SGA (see Methods for details) that shows the media used at each step and the markers under selection at each step (highlighted in red).

**Supplementary Figure 5. Ternary plot distance measurements identify mutants with altered cell cycle distributions. A.** Ternary plot diagrams of all haploid and diploid control strain observations; 292 and 219 observations are plotted for the haploid and diploid controls, respectively. **B.** (Left) Histogram of the theoretical distance distribution between the origin and two-dimensional points selected such that the x and y coordinates are selected from a normal distribution about 0 with a sigma of 1. (Middle and Right) Histograms of the distances in the ternary plots between the geometric median of the control strain observations and all individual control strain observations for haploid and diploid strains. Differences in the x-axis scale correspond to different sigma values for the theoretical and experimental distributions. **C.** A linear relationship in the quantile-quantile (q-q) plot demonstrates that the experimental distribution of distances of the control strain observations from the geometric median of these observations in the ternary plot matches the theoretical error model. The slope of the lines is the sigma value for the fitted distribution. **D.** Ternary plot of the median cell cycle distribution for each haploid mutant strain. **E.** (Top) Rank ordered distances between all haploid mutant strains and the geometric median of the control strain observations. (Bottom) The FDR values for the haploid mutant cell cycle distributions being different than the control strain distribution, which takes into account both changes in cell cycle distribution and the number of observations for that mutant (see Methods) **F.** Ternary plot of the median cell cycle distributions for each diploid mutant strain displayed as in panel D. **G.** Rank ordered distances and FDR values for all diploid mutant strains displayed as in panel E.

**Supplementary Figure 6. Genome-wide GCR screen. A.** YPD plates patches were generated by pinning strains from 96 well liquid plates using the Singer RoToR in triplicate. The 96 well plates contained the haploid progeny from a cross of RDKY8174 with the *S. cerevisiae* deletion collection. Patches were then replica plated onto GCR selection medium plates, and the number of papillae per patch were counted. Plate 4 from the cross is shown as an example. **B.** (Top) Histogram of the number of patches containing the specified number of papillae per patch from the three replicates of Plate 4 are plotted as bars, and the fitted normal distribution is displayed as red. (Middle) The p-values for each patch was calculated from the fitted normal distribution and plotted against the number of papillae per patch; dots are red if counts have a p-value < 0.01, blue otherwise. Each replicate is plotted separately, and lines connect the observations for each patch in each replicate; black dashed lines connect individual observations for mutations with at least one patch containing a significant number of papillae, whereas grey lines connect observations with no patches containing a significant number of papillae. (Bottom) The combined p-value (y-axis) was calculated by combining the p-values from each replica using Fisher’s sumlog procedure. X-axis positions are the average number of papillae in the three replicates. Labeled red points correspond to the mutation in strains with a significant combined p-value. **C.** Mutations causing larger GCR rates in the dGCR assay were more likely to be recovered in the initial screen. Red mutations were identified, and blue mutations were not. **D.** ROC curve analysis using 94 gold-standard positive mutations and 921 gold-standard negative mutations from previous studies demonstrates that the rescreening 620 strains using the average number of papillae per patch identifies mutations causing increased GCR rates. Youden index optimization implicates a threshold of the average number of papillae per patch at 1.8 times the average of the wild-type strain. **E.** Distribution of the average number of papillae per patch in the rescreen (black dots) with the 1.8xWT cutoff indicated (horizontal blue lines). The positions of mutations known to increase the dGCR rate are indicated as vertical red lines. The cutoff identifies 52 candidate mutations.

**Supplementary Literature Cited**

Ainsworth WB, Rome CM, Hjortso MA, Benton MG. 2012. Construction of a cytosolic firefly luciferase reporter cassette for use in pcr-mediated gene deletion and fusion in saccharomyces cerevisiae. Yeast. 29(12):505-517.

Basrai MA, Velculescu VE, Kinzler KW, Hieter P. 1999. Norf5/hug1 is a component of the mec1-mediated checkpoint response to DNA damage and replication arrest in saccharomyces cerevisiae. Mol Cell Biol. 19(10):7041-7049.

Benjamini Y, Hochberg Y. 1995. Controlling the false discovery rate: A practical and powerful approach to multiple testing. J Royal Statistical Society Series B. 57:289-300.

Benton MG, Glasser NR, Palecek SP. 2007. The utilization of a saccharomyces cerevisiae hug1p-gfp promoter-reporter construct for the selective detection of DNA damage. Mutat Res. 633(1):21-34.

Chabes A, Domkin V, Thelander L. 1999. Yeast sml1, a protein inhibitor of ribonucleotide reductase. J Biol Chem. 274(51):36679-36683.

Ciccia A, Elledge SJ. 2010. The DNA damage response: Making it safe to play with knives. Mol Cell. 40(2):179-204.

FlowJo. 2019. Flowjo software. Ashland, OR: Becton, Dickinson and Company.

Fox MH. 1980. A model for the computer analysis of synchronous DNA distributions obtained by flow cytometry. Cytometry. 1(1):71-77.

Greenall A, Lei G, Swan DC, James K, Wang L, Peters H, Wipat A, Wilkinson DJ, Lydall D. 2008. A genome wide analysis of the response to uncapped telomeres in budding yeast reveals a novel role for the nad+ biosynthetic gene bna2 in chromosome end protection. Genome Biol. 9(10):R146.

Hoose SA, Rawlings JA, Kelly MM, Leitch MC, Ababneh QO, Robles JP, Taylor D, Hoover EM, Hailu B, McEnery KA et al. 2012. A systematic analysis of cell cycle regulators in yeast reveals that most factors act independently of cell size to control initiation of division. PLoS Genet. 8(3):e1002590.

Huang M, Zhou Z, Elledge SJ. 1998. The DNA replication and damage checkpoint pathways induce transcription by inhibition of the crt1 repressor. Cell. 94(5):595-605.

Koren A, Soifer I, Barkai N. 2010. Mrc1-dependent scaling of the budding yeast DNA replication timing program. Genome Res. 20(6):781-790.

Lanz MC, Dibitetto D, Smolka MB. 2019. DNA damage kinase signaling: Checkpoint and repair at 30 years. EMBO J. 38(18):e101801.

Lee YD, Elledge SJ. 2006. Control of ribonucleotide reductase localization through an anchoring mechanism involving wtm1. Genes Dev. 20(3):334-344.

Lee YD, Wang J, Stubbe J, Elledge SJ. 2008. Dif1 is a DNA-damage-regulated facilitator of nuclear import for ribonucleotide reductase. Mol Cell. 32(1):70-80.

Li X, Jin X, Sharma S, Liu X, Zhang J, Niu Y, Li J, Li Z, Zhang J, Cao Q et al. 2019. Mck1 defines a key s-phase checkpoint effector in response to various degrees of replication threats. PLoS Genet. 15(8):e1008136.

Meurisse J, Bacquin A, Richet N, Charbonnier JB, Ochsenbein F, Peyroche A. 2014. Hug1 is an intrinsically disordered protein that inhibits ribonucleotide reductase activity by directly binding rnr2 subunit. Nucleic Acids Res. 42(21):13174-13185.

Moore SA. 1988. Kinetic evidence for a critical rate of protein synthesis in the saccharomyces cerevisiae yeast cell cycle. J Biol Chem. 263(20):9674-9681.

Popolo L, Vanoni M, Alberghina L. 1982. Control of the yeast cell cycle by protein synthesis. Exp Cell Res. 142(1):69-78.

Putnam CD, Allen-Soltero SR, Martinez SL, Chan JE, Hayes TK, Kolodner RD. 2012. Bioinformatic identification of genes suppressing genome instability. Proc Natl Acad Sci U S A. 109(47):E3251-3259.

Putnam CD, Jaehnig EJ, Kolodner RD. 2009. Perspectives on the DNA damage and replication checkpoint responses in saccharomyces cerevisiae. DNA Repair (Amst). 8(9):974-982.

Putnam CD, Srivatsan A, Nene RV, Martinez SL, Clotfelter SP, Bell SN, Somach SB, de Souza JE, Fonseca AF, de Souza SJ et al. 2016. A genetic network that suppresses genome rearrangements in saccharomyces cerevisiae and contains defects in cancers. Nat Commun. 7:11256.

Rouse J, Jackson SP. 2000. Lcd1: An essential gene involved in checkpoint control and regulation of the mec1 signalling pathway in saccharomyces cerevisiae. EMBO J. 19(21):5801-5812.

Soifer I, Barkai N. 2014. Systematic identification of cell size regulators in budding yeast. Mol Syst Biol. 10:761.

Spidlen J, Breuer K, Rosenberg C, Kotecha N, Brinkman RR. 2012. Flowrepository: A resource of annotated flow cytometry datasets associated with peer-reviewed publications. Cytometry A. 81(9):727-731.

Tsaponina O, Barsoum E, Astrom SU, Chabes A. 2011. Ixr1 is required for the expression of the ribonucleotide reductase rnr1 and maintenance of dntp pools. PLoS Genet. 7(5):e1002061.

Virtanen P, Gommers R, Oliphant TE, Haberland M, Reddy T, Cournapeau D, Burovski E, Peterson P, Weckesser W, Bright J et al. 2020. Scipy 1.0: Fundamental algorithms for scientific computing in python. Nat Methods. 17(3):261-272.

Weiszfeld E. 1937. Sur le point pour lequel la somme des distrandes de n points donnes est minimum. Tohoku Mathematical Journal 43:355-386.

Wu X, Huang M. 2008. Dif1 controls subcellular localization of ribonucleotide reductase by mediating nuclear import of the r2 subunit. Mol Cell Biol. 28(23):7156-7167.

Zhang Z, Yang K, Chen CC, Feser J, Huang M. 2007. Role of the c terminus of the ribonucleotide reductase large subunit in enzyme regeneration and its inhibition by sml1. Proc Natl Acad Sci U S A. 104(7):2217-2222.

Zhao X, Chabes A, Domkin V, Thelander L, Rothstein R. 2001. The ribonucleotide reductase inhibitor sml1 is a new target of the mec1/rad53 kinase cascade during growth and in response to DNA damage. EMBO J. 20(13):3544-3553.

Zhao X, Muller EG, Rothstein R. 1998. A suppressor of two essential checkpoint genes identifies a novel protein that negatively affects dntp pools. Mol Cell. 2(3):329-340.
