# Supplementary material for: Identification of different classes of genome instability suppressor genes through analysis of DNA damage response markers
Source: G3 (Bethesda). 2024 Mar 25;14(6):jkae064. doi: 10.1093/g3journal/jkae064 (PMC11152081; doi:10.1093/g3journal/jkae064)
Supplement: jkae064_Supplementary_Data [file jkae064_supplementary_data.zip › Supplementary_Table_6_G3-2024-404884.docx]

**Table S6. GCR rates of reconstructed strains.**

| **Genotype** | **dGCR rate (reconstructed strain)** |
| --- | --- |
| Wild-type* | 8.1 [6.4-15] x10^-8^ (1) |
| *rpl43aΔ* | 1.18 [0.76-1.43] x10^-7^ (1.45) |
| *hof1Δ* | 6.57 [3.99-13.5] x10^-8^ (0.81) |
| *cnm67Δ* | 1.08 [0.00-2.28] x10^-7^ (1.34) |
| *lip2Δ* | 4.61 [2.06-13.1] x10^-7^ (5.69) |
| *imp21Δ* | 1.33 [1.1-1.6] x10^-7^ (2.01) |
| *mrm1Δ* | 9.14 [0.59-20] x10^-7^ (11.3) |

*wildtype rates from Nene and Putnam2009
