# Supplementary figures and images for: Identification of different classes of genome instability suppressor genes through analysis of DNA damage response markers
Source: G3 (Bethesda). 2024 Mar 25;14(6):jkae064. doi: 10.1093/g3journal/jkae064 (PMC11152081; doi:10.1093/g3journal/jkae064)

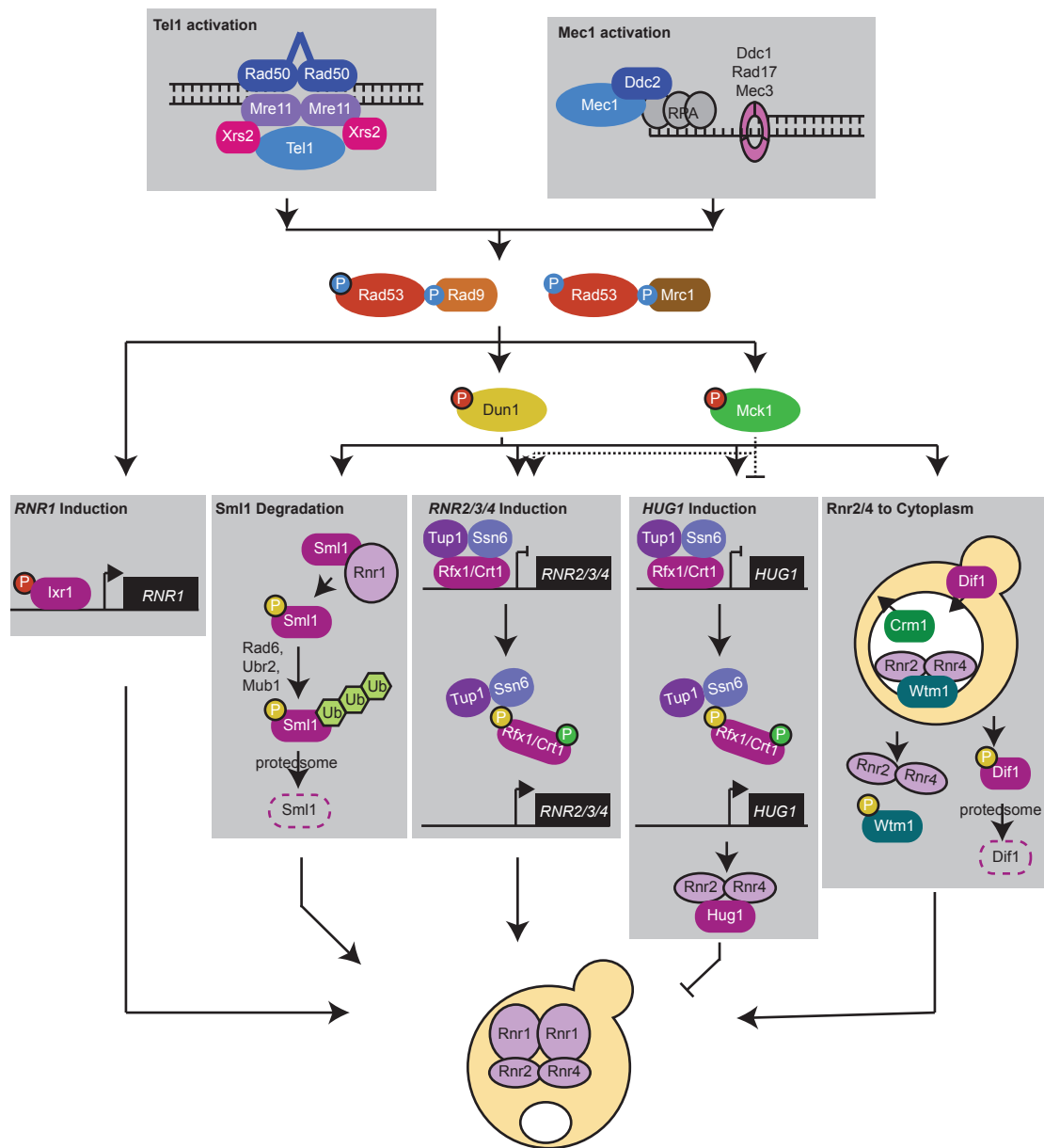

Supplement: jkae064_Supplementary_Data [file jkae064_supplementary_data.zip › Supplementary_Figure_1_G3-2024-404884.pdf]

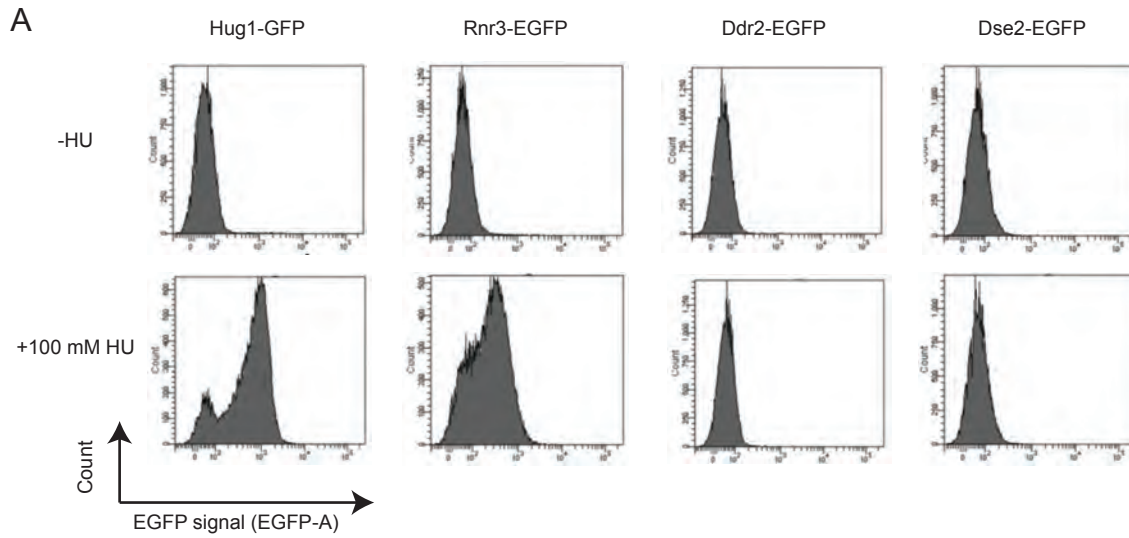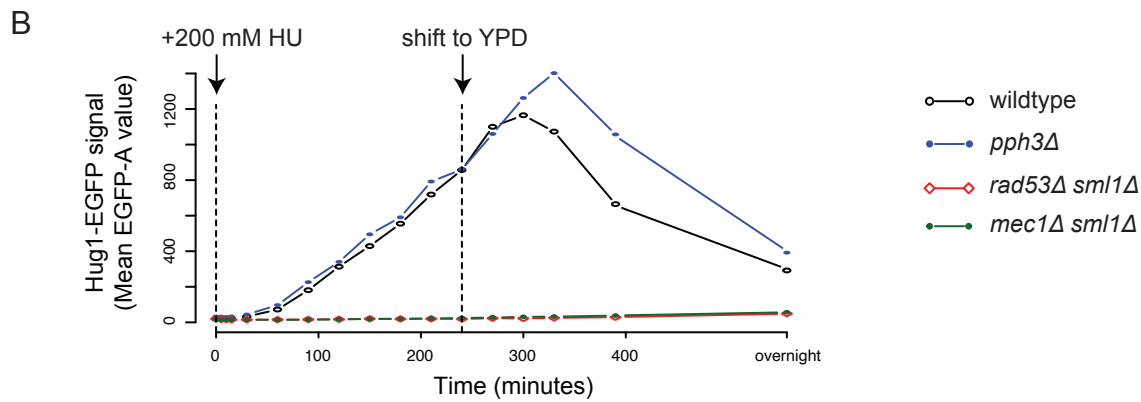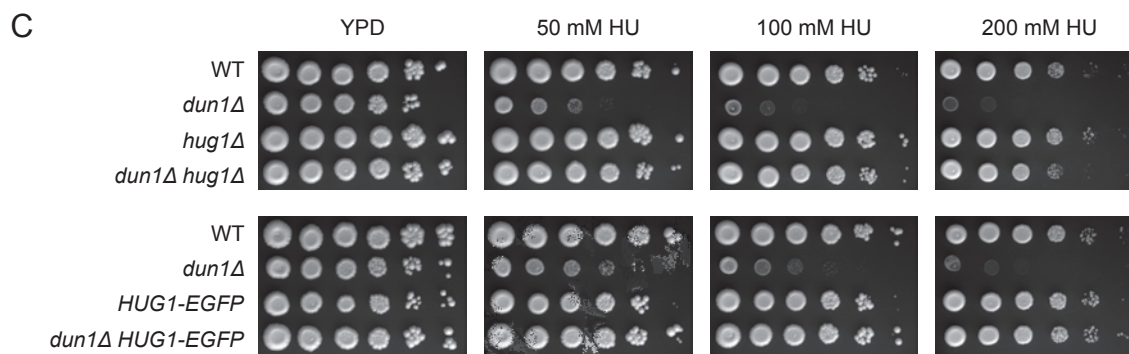

Supplement: jkae064_Supplementary_Data [file jkae064_supplementary_data.zip › Supplementary_Figure_2_G3-2024-404884.pdf]

A

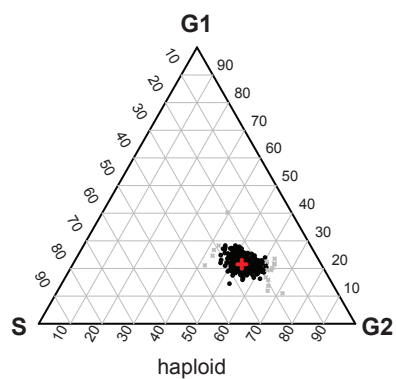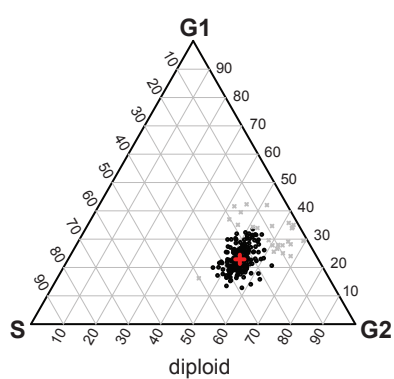

C

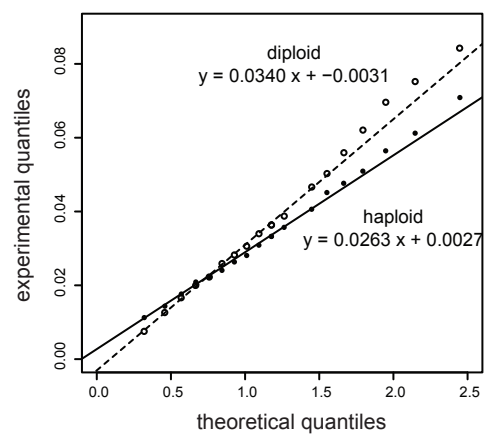

B

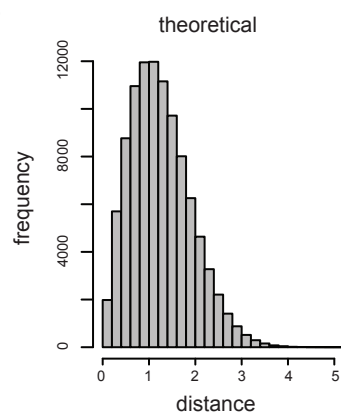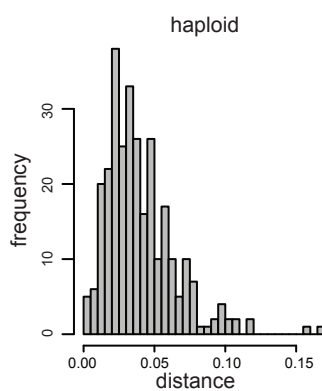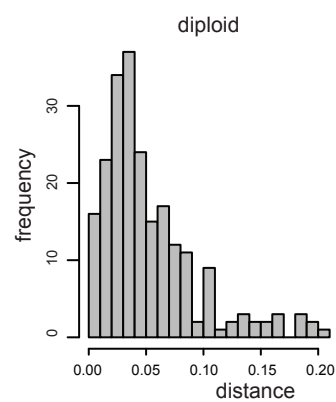

D

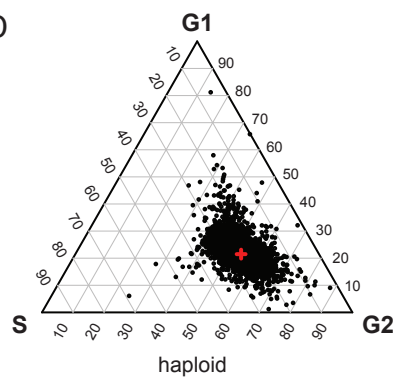

E

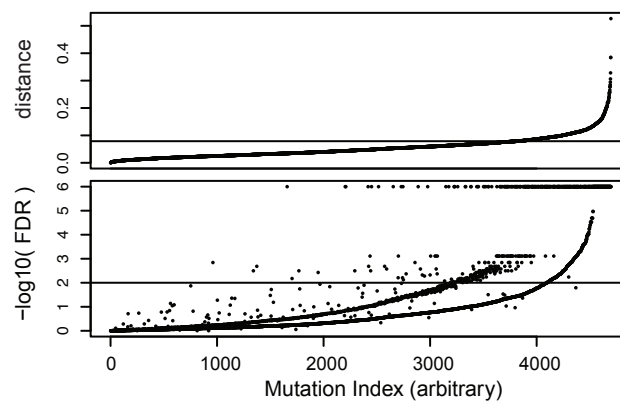

F

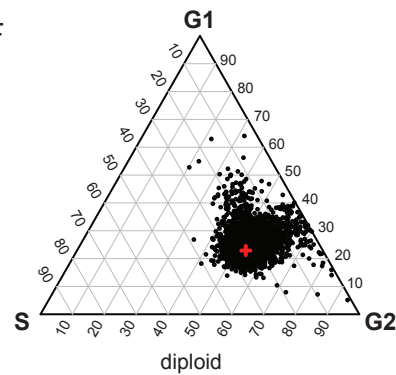

G

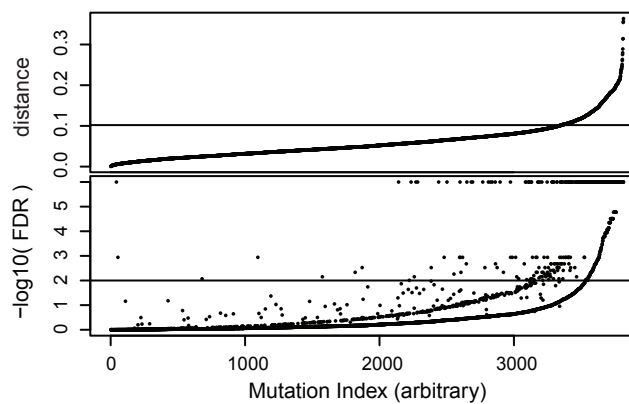

Supplement: jkae064_Supplementary_Data [file jkae064_supplementary_data.zip › Supplementary_Figure_5_G3-2024-404884.pdf]

A

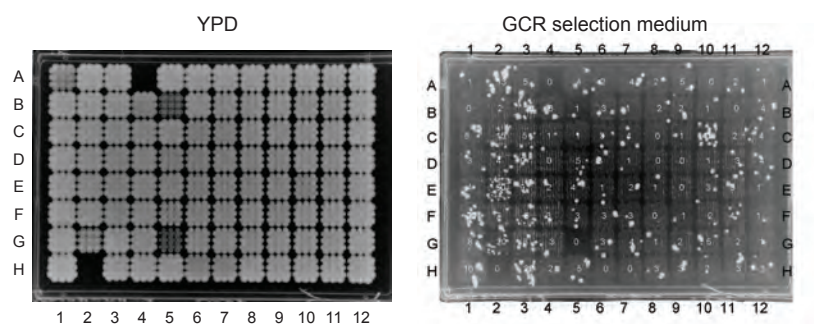

B

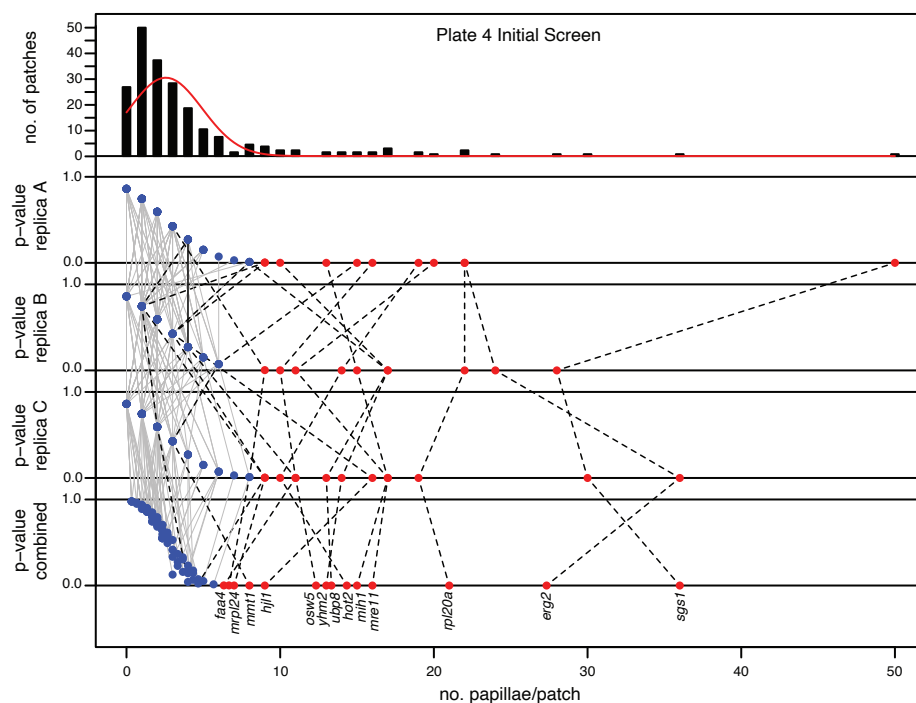

C

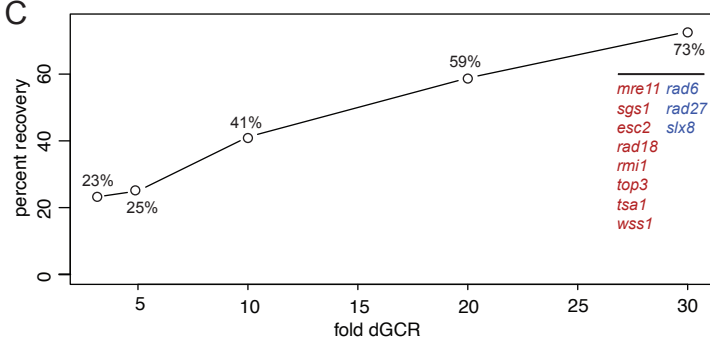

D

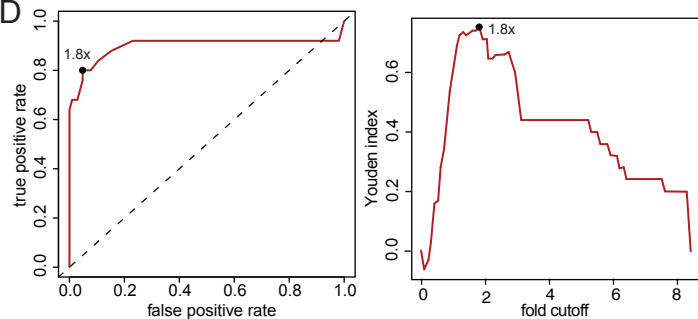

E

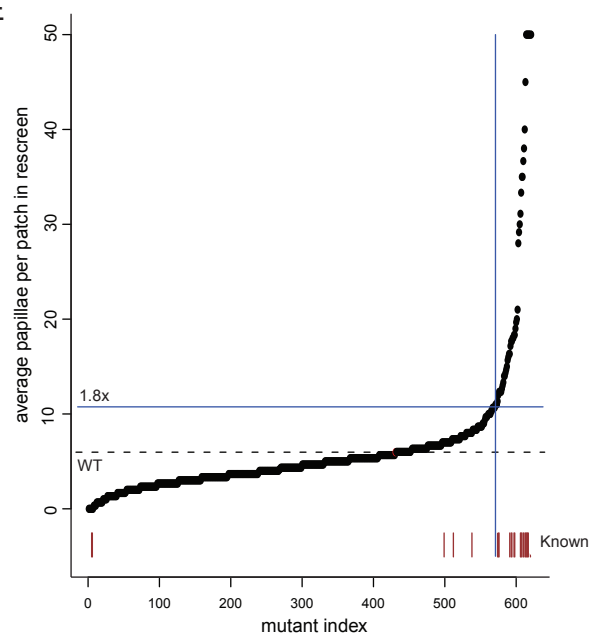

Supplement: jkae064_Supplementary_Data [file jkae064_supplementary_data.zip › Supplementary_Figure_6_G3-2024-404884.pdf]
